# Analysis of Pharmacological Activities and Mechanisms of Essential Oil in Flowers of *Citrus grandis* ‘Tomentosa’ by GC-MS/MS and Network Pharmacology

**DOI:** 10.3390/cimb47070541

**Published:** 2025-07-11

**Authors:** Danxi Yan, Shuyi Wen, Mingxia Chen, Jinlan Huang, Guihao Zhang, Renkai Li, Jiamin Lu, Zhongxuan Yao, Fei Gao, Jieshu You

**Affiliations:** 1 School of Pharmacy, Shenzhen University Medical School, Shenzhen University, Shenzhen 518055, China; yandanxi@163.com (D.Y.); wsya1009@163.com (S.W.); chenmingxia2023@163.com (M.C.); 2 College of Pharmacy, Shenzhen Technology University, Shenzhen 518118, China; hjl202101101106@163.com (J.H.); zgh202101101026@163.com (G.Z.); rrkli@connect.hku.hk (R.L.); chln372111982023@163.com (J.L.); 13827595062@163.com (Z.Y.); 3 Department of Pharmacology and Pharmacy, Li Ka Shing Faculty of Medicine, The University of Hong Kong, Hong Kong 518000, China; 4 College of Pharmacy, Chengdu University of Traditional Chinese Medicine, Chengdu 610072, China; feigao207@yeah.net

**Keywords:** flowers of *Citrus grandis* ‘Tomentosa’, essential oils, GC-MS/MS, network pharmacology, active component–target–disease

## Abstract

According to our research, the flowers from *Citrus grandis* ‘Tomentosa’ contain rich biologically active essential oil components, but the chemical components and relative pharmacological properties have not been systematically studied. Therefore, the study aimed to identify the essential oil components by GC-MS/MS and explore the pharmacological activity and mechanism of these essential oil components by a network pharmacology approach. Finally, GC-MS/MS analysis identified 43 essential oil components, which corresponded to 739 potential targets. GO analysis results showed that 12, 18, and 12 entries were related to biological processes, cellular components, and molecular functions, respectively. A total of 120 pathways were obtained based on KEGG analysis, of which the most important was the adenylate cyclase-inhibiting G protein-coupled acetylcholine receptor signaling pathway. The “active component–target–disease” network further demonstrated these essential oil components’ potential efficacy against pain, tumors, neuropsychiatric diseases, eye diseases, and respiratory diseases, which were highly related to PPARA, GABRA1, PTGS2, and SLC6A2. Experimental validation confirmed that β-caryophyllene, a major constituent, dose-dependently inhibited the proliferation of HT29 and MCF-7 cells (0–320 μM). This study provides a reliable basis for elucidating the pharmacological activity of the essential oil components and related mechanisms, which is beneficial to the comprehensive utilization and development of *Citrus grandis* ‘Tomentosa’.

## 1. Introduction

With growing global interest in green medicine, functional foods and traditional Chinese medicines (TCM) have become a promising approach for preventing and treating diseases. Citri Grandis Exocarpium (Huajuhong), derived from the fruit epicarp of *Citrus grandis* ‘Tomentosa’ or *Citrus grandis* (L.) Osbeck native to Huazhou town in Guangdong province, southern China, has been documented since the Ming Dynasty as both a medicinal and edible resource. According to the theory of TCM, Citri Grandis Exocarpium is acrid, bitter, and warm, and acts on the lung and spleen channels. It can rectify qi, dry dampness, and dissolve phlegm. Modern research found that flavonoids, essential oil, polysaccharides, coumarins, and limonoids are the main active components of Citri Grandis Exocarpium, and its main pharmacological effects have expanded to anti-oxidant, anti-inflammatory, anti-microbial, anti-tussive, anti-proliferative, and anti-atherosclerotic activities [1,2,3,4]. The essential oil components such as limonene, laurylene, terpenes, and caryophyllene in Citri Grandis Exocarpium have been proven to have expectorant, cough-relieving, analgesic, bactericidal, and anti-inflammatory effects [4,5]. Actually, some modern studies have also demonstrated that there are also rich essential oil components in the flowers and leaves of *Citrus grandis* ‘Tomentosa’ [6,7]. Qi et al. [7] found that the components and contents of essential oil were similar between the flowers and fruits of *Citrus grandis* ‘Tomentosa’, which is beneficial for promoting an understanding of the medicinal value and development of *Citrus grandis* ‘Tomentosa’. However, there are few studies on the relevant pharmacological activities associated with the chemical compounds in the flower of *Citrus grandis* ‘Tomentosa’.

Gas chromatography coupled with tandem mass spectrometry (GC-MS/MS) is a predominant tool for identifying and analyzing essential oil components. Compared with gas chromatography-mass spectrometry (GC-MS), GC-MS/MS technology can perform a secondary cleavage of the target substance, effectively reducing complex sample matrix interference while enhancing detection sensitivity and accuracy [8]. Network pharmacology, proposed by Andrew L Hopkins [9], has the characteristics of integrity and systematicity, which are highly consistent with the theories of “holistic concept” and “syndrome differentiation and treatment” in TCM, as well as the functional characteristics of “multi-components, multi-pathways, and multi-targets” in Chinese medicine prescriptions, so the network pharmacology has been widely applied in modern research of TCM [10,11].

In the present study, essential oil components in the flowers of *Citrus grandis* ‘Tomentosa’ were deciphered by GC-MS/MS and network pharmacology was adopted to construct the “active component–target–disease” network to explore the potential pharmacological activities and mechanism. In addition, a simple validation of the role of the important compound β-caryophyllene in HT29 and MCF-7cells was conducted. This study would help reduce resource waste and generate economic benefits.

## 2. Materials and Methods

### 2.1. Preparation of Flowers of Citrus grandis ‘Tomentosa’

**Figure 1:**
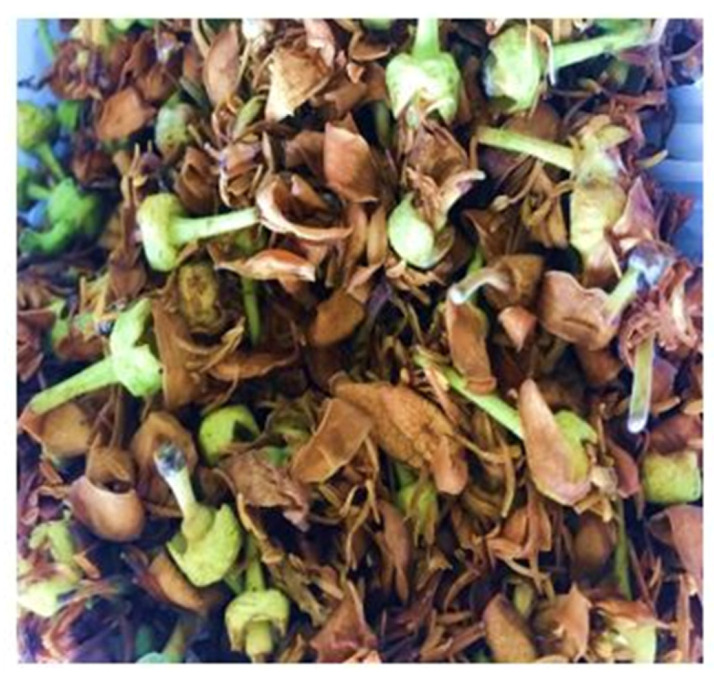
Flowers of *Citrus grandis* ‘Tomentosa’.

Flowers of *Citrus grandis* ‘Tomentosa’ (Figure 1) were collected from Huazhou city in March 2023 (Voucher Number: 20230302) and were authenticated by Professor Huan-lan Liu from Guangdong University of Chinese Medicine. A total of 50 g of flowers was extracted in 500 mL of water for 18 h via a steam distillation method. The extract was dried by concentrating and freeze-drying. The sample was adjusted to a volume of 1 mL using n-hexane and passed through a 0.22 μm PTFE syringe filter.

### 2.2. Identification of Essential Oil Components

The extract was analyzed with an Agilent 5977B GC-MS/MS system to identify the essential oil in the flowers of *Citrus grandis* ‘Tomentosa’. The equipment has a HP-5 ms column (30 m × 0.25 mm i.d., 0.25 μm film thickness). The carrier gas was helium at a constant flow rate of 1.0 mL/min. The sample injection was carried out in a split ratio of 5:1 and an injection volume of 1 µL. The oven was operated as follows: an initial temperature of 60 °C for 3 min, heating from 60 °C to 110 °C at a rate of 5 °C/min, and further increased to 150 °C at a rate of 4 °C/min, where the final temperature of 240 °C was held for 5 min. The mass spectrometer was operated in electron ionization mode at 70 eV, with an ion source temperature at 230 °C and quadrupole temperature at 150 °C. The mass scan range was 35 to 550 amu.

### 2.3. Target Screening of Essential Oil Components

The protein targets of the essential oil composition in flowers of *Citrus grandis* ‘Tomentosa’ were screened through the Traditional Chinese Medicine Systems Pharmacology Database and Analysis Platform (TCMSP, https://tcmsp-e.com/tcmsp.php (accessed on 12 December 2024)), STITCH (http://stitch.embl.de/ (accessed on 12 December 2024)), SwissTargetPrediction (http://www.swisstargetprediction.ch/ (accessed on 12 December 2024)), and Similarity Ensemble Approach (SEA, http://sea.bkslab.org/ (accessed on similarity ensemble approach)) for each chemical component. Homo sapiens were the only species for the targets and the repetitive targets were removed. Cytoscape software (Version 3.9.1) was adopted for constructing the component–target network of the essential oil components.

### 2.4. GO Functional and KEGG Pathway Analysis

GO enrichment and KEGG analysis were conducted through the WEB-based Gene SeT AnaLysis Toolkit (WebGestalt, www.webgestalt.org (accessed on 18 December 2024)) 2019. GO enrichment analysis was used to examine the enrichment of GO entries in gene sets, which could help to understand the common characteristics of genes in a gene set in terms of biological processes, molecular functions, and cellular composition [12]. KEGG pathway analysis was used for the systematic analysis of gene function, linking gene lists with higher-order functional information to receive significantly enriched biological information [13]. The GO and KEGG pathway enrichment analyses were carried out for the top 10 hub genes with a threshold of FDR < 0.05.

### 2.5. Network Construction of Active Component–Target–Disease

TTD (Therapeutic Target Database, http://db.idrblab.net/ttd/ (accessed on 20 December 2024)) is a database to provide information about the known and explored therapeutic protein and nucleic acid targets, the targeted disease, pathway information, and the corresponding drugs [14]. Diseases were obtained from TTD based on the successful clinical trial targets via PPI network analysis. Then the component–target–disease network was constructed using Cytoscape software (Version 3.9.1).

### 2.6. Cell Lines and Cell Culture

Human breast cancer MCF-7 cells and human colon cancer HT29 cells were purchased from the American Type Culture Collection. MCF-7 cells are widely utilized in tumor biology and hormone action mechanism studies [15]. HT29 cells exhibit significant applications in colon cancer and toxicology research [16]. These cells were cultured in DMEM medium supplemented with 10% fetal bovine serum (FBS, Gibco Life Technologies, Lofer, Austria) in a humidified incubator at 37 °C supplied with 5% CO_2_.

### 2.7. Cell Proliferation Assay

As the relative amount (RA)% of β-caryophyllene was relatively high in the flowers of *Citrus grandis* ‘Tomentosa’, the effects of β-caryophyllene on cancer cell proliferation were detected by MTT assay. β-caryophyllene was purchased from Sigma-Aldrich, Inc., (St. Louis, MO, USA) and was dissolved in DMSO and 5% Tween 80 in distilled water (*v*/*v*) for in vitro study. Briefly, MCF-7 and HT29 cells were cultured in 96-well plates for 48 h. Then, different concentrations of β-caryophyllene (6–320 µM) and doxorubicin (DOX, 0–10 µM) were added to these cancer cells and incubated for 48 h. Then MTT reagent was added to each well and incubated for 4 h at 37 °C. A triplicate independent experiment was conducted. The viable cells converted MTT into formazan, which dissolved in DMSO to generate a blue-purple color. The absorbance was measured at 570 nm using a Model 680 Microplate Reader. (Bio-Rad Laboratories, Hercules, CA, USA). The relative percentage of cell survival was calculated by dividing the absorbance of treated cells by the control in each experiment.

### 2.8. Statistical Analysis

All quantitative data were presented as the means ± standard deviation (SD). The significance values were analyzed by SPSS 25.0 software using homogeneity of variance and a one-way analysis of variance (ANOVA). Normality was first assessed using the Shapiro–Wilk test (*p* > 0.05 for all datasets). For homogeneous variances (confirmed via the Brown–Forsythe test), a one-way ANOVA with Tukey’s post hoc test was performed. Non-parametric data were analyzed with a Kruskal–Wallis test followed by Dunn’s correction. GraphPad Prism 8.0 software (GraphPad Software Inc., San Diego, CA, USA) was used to plot the histogram for protein expression analysis. A *p*-value < 0.05 was considered statistically significant.

## 3. Results

### 3.1. Determination of Essential Oil Components in Flowers of Citrus grandis ‘Tomentosa’

**Figure 2:**
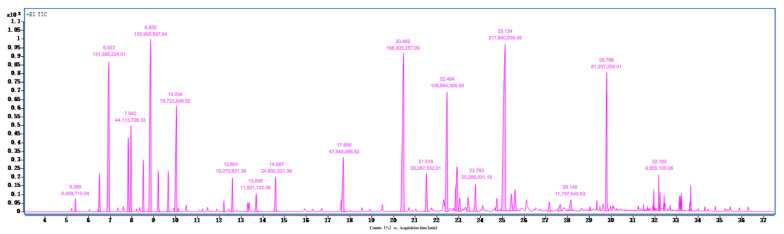
GC-MS chromatogram obtained from the essential oil in flowers of *Citrus grandis* ‘Tomentosa’.

A total of 0.46 g essential oil components was obtained from the flowers of *Citrus grandis* ‘Tomentosa’, representing an extraction yield of 0.92% (the experiment was independently repeated three times, with the results expressed as mean values). The total ion chromatogram of essential oil components from the flowers of *Citrus grandis* ‘Tomentosa’ by GC-MS/MS was shown in Figure 2. A total of 43 essential oil chemical components were deciphered and listed in Table 1. These essential oils were mainly terpenes (54.17%), including β-caryophyllene (11.42%), γ-terpinene (9.23%), myrcene (8.93%), and (−)-germacrene D (7.40%), and alcohols (31.17%), mainly including nerolidol (15.61%), 3,7-dimethylocta-1,6-dien-3-ol (5.35%), and farnesol (5.52%). It is noted that the measured values are not definitive and may exhibit variations influenced by seasonal factors.

### 3.2. Potential Targets of Essential Oil Components

**Figure 3:**
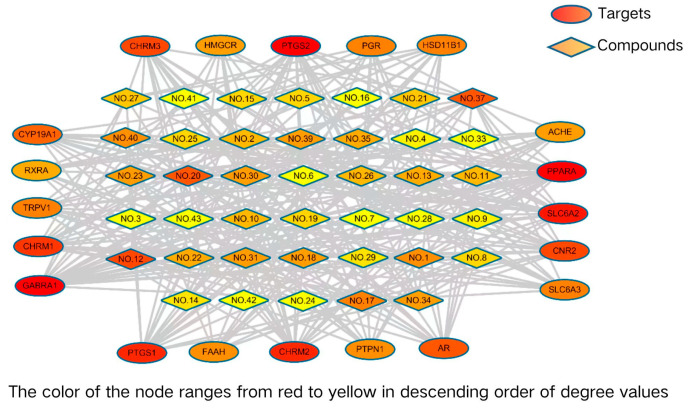
The component–main target network of essential oil compositions. Number of nodes: 60, number of edges: 316, network diameter: 4, and network density: 0.179.

A total of 2024 potential targets of all essential oil components were obtained from the databases of TCMSP, SwissTargetPrediction, STITCH, and SEA with the screening criteria “Homo. Sapiens” after deleting duplicate values (Supplementary File, Table S1). Figure S1 shows the “component–target” network of essential oil compositions in flowers of *Citrus grandis* ‘Tomentosa’, which contained 739 targets. The screening criteria of candidate targets were “high” for “degree” (twice higher than the median value), “betweenness centrality”, and “closeness centrality” (higher than the median value), and “lower” for “average shortest path length” (lower than the median value). Ultimately, it was found that 20 direct target proteins were highly correlated with these essential oil compositions, among which peroxisome proliferator-activated receptor alpha (PPARA), gamma-aminobutyric acid receptor subunit alpha-1 (GABRA1), prostaglandin G/H synthase 2 (PTGS2), and sodium-dependent noradrenaline transporter (SLC6A2) were associated with 26, 25, 22, and 21 chemical components, respectively (Table 2, Figure 3).

### 3.3. Gene Ontology (GO) Function and Kyoto Encyclopedia of Genes and Genomes (KEGG) Pathway Analysis

**Figure 4:**
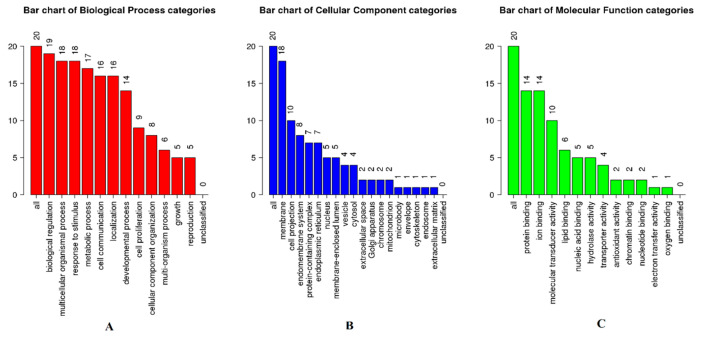
GO analysis of target proteins: (**A**) biological process category, (**B**) cellular component category, and (**C**) molecular functions category. The abscissa represents the GO entry name, and the ordinate represents the number and percentage of proteins corresponding to the entry.

For the analysis of GO function, the most significant items in biological process categories were biological regulation, multicellular organismal processes, and response to stimulus (Figure 4A). In terms of cellular component categories, the target proteins mainly involved membrane, cell projection, and the endomembrane system (Figure 4B). For molecular function categories, the target proteins were mainly enriched in protein binding, ion binding, and molecular transducer activity (Figure 4C).

**Figure 5:**
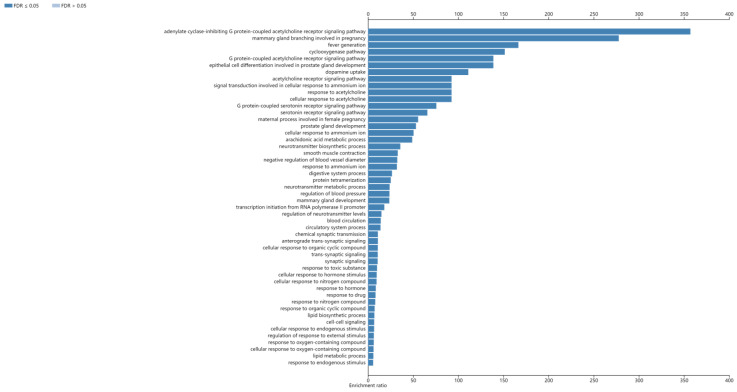
Enriched KEGG pathway of potential targets. The horizontal axis represents the enrichment ratio, while the vertical axis displays KEGG pathways.

These proteins were found to participate in a total of 127 signaling pathways, which were mainly associated with the adenylate cyclase-inhibiting G protein-coupled acetylcholine receptor signaling pathway, mammary gland branching involved in pregnancy, and fever generation (Figure 5).

### 3.4. Network Construction of Active Component–Target–Disease

The diseases obtained by TTD were shown in Table 3. These diseases could be divided into pain, tumors, neuropsychiatric diseases, ophthalmic diseases, and respiratory system diseases. Among them, pain mainly included chronic pain, acute pain, musculoskeletal pain, visceral spasms, and dysmenorrhea. Tumors were mainly involved in acute myeloid leukemia, hormonally-responsive breast cancer, Kaposi sarcoma, and cutaneous T-cell lymphoma. Neuropsychiatric diseases mainly included attention deficit hyperactivity disorder, anesthesia, and major depressive disorder. Ophthalmic diseases mainly involved glaucoma/ocular hypertension, miosis, and ophthalmic surgery injury. Respiratory system diseases mainly included respiratory distress syndrome, asthma, and obstructive lung disease.

**Figure 6:**
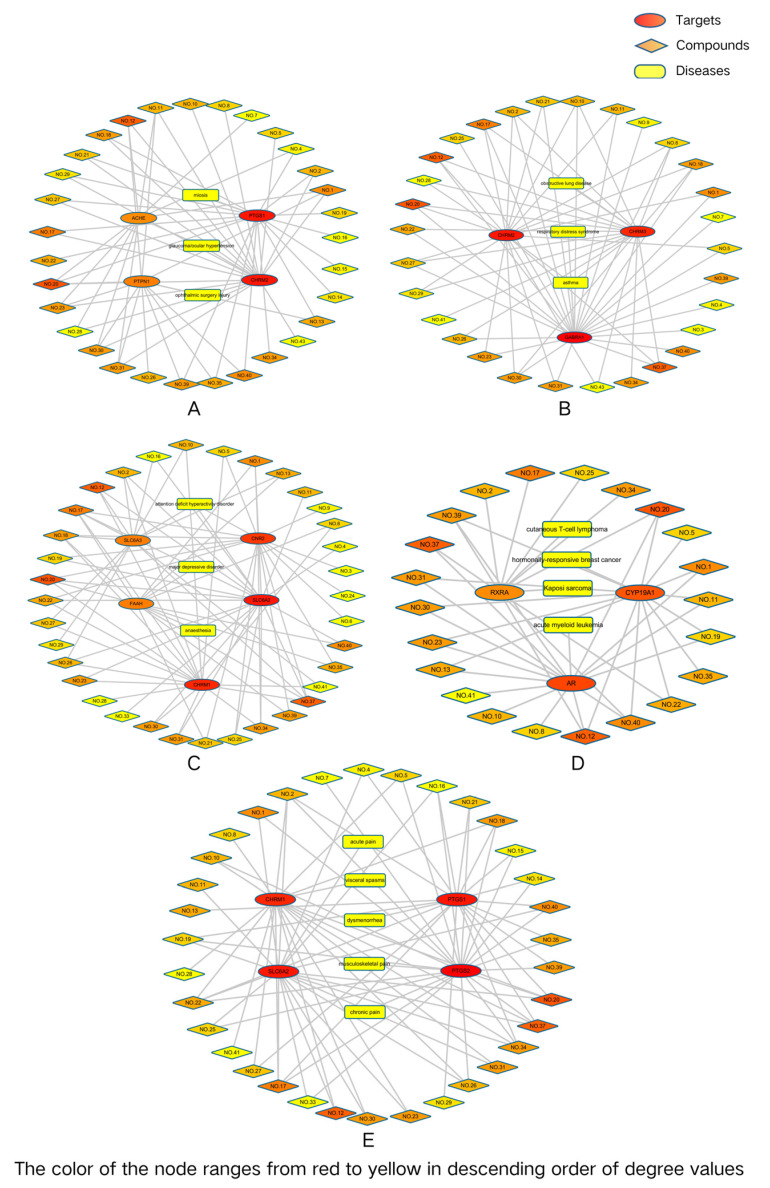
The component–target–disease networks. (**A**) Component–target–pain network, number of nodes: 43, number of edges: 83, network diameter: 4, and network density: 0.092. (**B**) Component–target–tumor network, number of nodes: 29, number of edges: 43, network diameter: 4, and network density: 0.106. (**C**) Component–target–neuropsychiatric disease network, number of nodes: 43, number of edges: 83, network diameter: 4, and network density: 0.092. (**D**) Component–target–ophthalmic diseases network, number of nodes: 38, number of edges: 62, network diameter: 4, and network density: 0.088. (**E**) Component–target–respiratory system disease network, number of nodes: 36, number of edges: 65, network diameter: 4, and network density: 0.103.

“Active component–target–disease” networks for pain, tumors, neuropsychiatric diseases, ophthalmic diseases, and respiratory system diseases were constructed (Figure 6). As shown in Figure 6A, six essential oil components could be associated with chronic pain, acute pain, musculoskeletal pain, visceral spasms, and dysmenorrhea by acting on four protein targets, including CHRM1, PTGS1, PTGS2, and SLC6A2. These six essential oil components were (−)-β-pinene, α-selinene, α-muurolene, β-caryophyllene, farnesol, and dibutyl phthalate. In Figure 6B, α-selinene, methyl linoleate, α-terpineol, and methyl linolenate were associated with acute myeloid leukemia, hormonally-responsive breast cancer, Kaposi sarcoma, and cutaneous T-cell lymphoma by acting on the RXRA, CYP19A1, and AR. As shown in Figure 6C, β- caryophyllene and dibutyl phthalate could directly act on CHRM1, FAAH, CNR2, SLC6A2, and SLC6A3, which were associated with anesthesia, attention deficit hyperactivity disorder, and major depressive disorder. Figure 6D shows the network of “active component–target–ophthalmic disease”. α-Selinene could act on multiple protein targets, including ACHE, PTPN1, PTGS1, and CHRM2, and were further associated with miosis, glaucoma/ocular hypertension, and ophthalmic surgery injury. In addition, 18 essential oil components such as α-terpineol, α-selinene, and β-caryophyllene could act on CHRM2 and be linked to glaucoma/ocular hypertension. A total of 18 essential oil components such as β-caryophyllene, α-selinene, farnesol, methyl linoleate, and methyl linolenate could be connected to miosis by acting on PTGS1. In Figure 6E, 10 essential oil components could act on multiple protein targets, including CHRM2, CHRM3, and GABRA1, which were further associated with obstructive lung disease, asthma, and respiratory distress syndrome. These 10 volatile oil components were 2-furanmethanol,5-ethenyltetrahydro-a,a,5-trimethyl-, (2R,5S)-rel-, α-terpineol, β-caryophyllene, (-)-β-pinene, 3,7-dimethylocta-1,6-dien-3-ol, α-caryophyllene, dibutyl phthalate, (-)-tau-muurolol, spathulenol, and α-Selinene.

### 3.5. Effect of β-Caryophyllene on Cell Proliferation in HT29 and MCF-7cells

**Figure 7:**
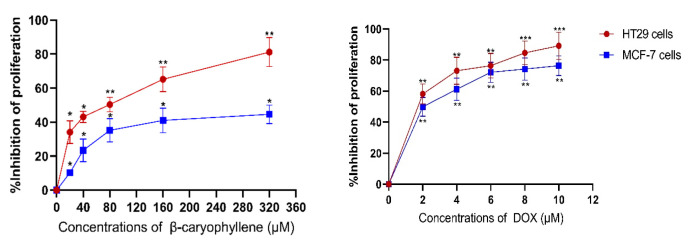
Dose-dependent anti-proliferative effect of β-caryophyllene and DOX on HT-29 and MCF-7 cell lines assessed by MTT-assay (values were represented as mean ± SD, *n* = 3). * *p* < 0.05, ** *p* < 0.01, and *** *p* < 0.001 compared to control group (0 μM).

The anti-proliferative properties of β-caryophyllene and the positive control doxorubicin (DOX) in HT29 and MCF-7 cells was assessed by an MTT assay. As shown in Figure 7, 48 h β-caryophyllene and DOX treatment dose-dependently inhibited the proliferation of HT29 and MCF-7cells.

## 4. Discussion

Over the past decade, network pharmacology has achieved remarkable advancements in facilitating the analysis of the multiple components and action mechanisms of TCM. This study integrated network pharmacology with GC-MS/MS to characterize the essential oil from the flowers from *Citrus grandis* ‘Tomentosa’ and construct an “active component–target–diseases” network.

The terpenes and alcohols (the oxygen-containing derivatives) were determined as the main essential oil components in the flowers of *Citrus grandis* ‘Tomentosa’ by GC-MS/MS, which is similar to previous reports [4,7]. The components in our results were not completely consistent with those reported in other studies, which may be due to differences in extraction methods and batches. The terpenes are hydrocarbons composed of isoprene units found present in essential oils and have broad bioactivities such as anti-inflammatory, anti-bacterial, and anti-oxidant effects [17,18,19,20]. The contents of terpenes extracted from the flowers of *Citrus grandis* ‘Tomentosa’ were higher than those extracted from the *Citri grandis* Exocarpium (fruit) and the leaves of *Citrus grandis* ‘Tomentosa’ [4,6]. β-caryophyllene is a naturally occurring sesquiterpene found in numerous essential oils. It is reported that β-caryophyllene exerts significant neuroprotective, anti-inflammatory, antibiotic, anti-oxidant and anti-cancer effects [21,22,23]. In this study, β-caryophyllene was also demonstrated to have the effect of inhibiting the proliferation of HT29 and MCF-7cells. Jean et al. [24] demonstrated that β-caryophyllene significantly increased the anticancer activity of alpha-humulene and isocaryophyllene on MCF-7 cells. Many other studies also found that many essential oil compositions rich in β-caryophyllene exhibit significant inhibitory effects on the proliferation of HT29 and MCF-7 cells.

The results of network pharmacology indicated that a total of 43 essential oil components in the flowers of *Citrus grandis* ‘Tomentosa’ corresponded to 2024 target proteins across the TCMSP, Swiss Target Prediction, STITCH, and SEA databases. It was found that there were 20 direct targets highly correlating with the essential oil components after screening according to the specified conditions, among which PPARA, GABRA1, PTGS2, and SLC6A2 were associated with 26, 25, 22, and 21 chemical components, respectively. GO functional enrichment analysis showed that 12, 18, and 12 GO entries were involved in biological processes, cellular components, and molecular functions, respectively. A total of 127 KEGG pathways were identified, with the most important being the adenylate cyclase-inhibiting G protein-coupled acetylcholine receptor signaling pathway. TTD analysis obtained a total of 33 diseases, predominantly categorized as pain, tumors, neuropsychiatric diseases, ophthalmic diseases, and respiratory system diseases.

In these KEGG pathways, the generation of fever and the cyclooxygenase pathway were involved in the regulation of the four diseases. Fever is a well-coordinated pathophysiological phenomenon related to infection and trauma, characterized by a body temperature above normal [25,26]. Fever is considered beneficial to some extent, as an increase in body temperature enhances the activity of immune factors and cells. Elevated body temperature can cause an increase in the bactericidal activity of neutrophils and macrophages, T cell proliferation and differentiation, B cell proliferation, antibody production, and the stimulation of acute phase protein synthesis [27,28,29]. Meanwhile, fever can damage the replication of many microorganisms [30]. Many prospective and retrospective studies have shown that febrile infections can reduce the risk of cancer and may be closely related to the spontaneous remission of various tumors [31]. Cyclooxygenase (COX) is a key enzyme in the synthesis of various prostaglandins, with two isoenzymes, namely constitutive enzyme (COX-1) and inducible enzyme (COX-2). The two forms of COX isoenzymes are closely related to the functions of various systems in the body, including digestion, nerves, circulation, urology, and reproduction, and also play an important role in the occurrence and treatment of inflammation and certain cancers [32,33,34].

“Active components–target–diseases”, “component–target–pain” “component–target–tumor”, “component–target–neuropsychiatric diseases”, “component–target–ophthalmic diseases”, and “component–target–respiratory system diseases” networks were established. In these, SLC6A2 was considered as the main target because there were 19 volatile oil components acting on it and it was highly associated with pain and neuropsychiatric disorders. The SLC6A2 gene encodes the neurotransmitter sodium ion symporter family, which is a multi-channel membrane protein responsible for the reuptake of norepinephrine into presynaptic nerve endings and is a regulator of norepinephrine homeostasis [35]. In addition, mutations in the SLC6A2 gene can lead to orthostatic intolerance, a syndrome characterized by dizziness, fatigue, altered consciousness, and syncope [36]. Modern research has also found that SLC6A2 is associated with heart failure, depression, and hypertension [37,38]. α-Selinene was considered to be the most active essential oil component in the flowers of *Citrus grandis* ‘Tomentosa’, as it involved acute pain, chronic pain, musculoskeletal pain, cutaneous T-cell lymphoma, hormonally-responsive breast cancer, Kaposi sarcoma, acute myeloid leukemia, attention deficit hyperactivity disorder, major depressive disorder, anesthesia, miosis, glaucoma/ocular hypertension, ophthalmic surgery injury, asthma, respiratory distress syndrome, and obstructive lung disease. Selinene belongs to the sesquiterpenoid compound and its odor is like the taste of soil and plant roots. Studies have found that selinene is the main component of sedge and it also comes from other plants such as black pepper, ginger, celery seeds, sedge, cloud wood incense, and Taiwan red juniper. Pharmacological studies have found that it has characteristics of anti-spasticity, pain relief, balancing the central nervous system, and assisting in sedation [39].

While this study successfully constructed a “component–target–disease” network, the predicted potential targets remain to be experimentally validated, which is a limitation of the current work. In follow-up studies, cellular and animal experiments will be performed to further verify the reliability of these findings.

## 5. Conclusions

This study attempted to explore the pharmacological activity and mechanism of the essential oil in flowers of *Citrus grandis* ‘Tomentosa’ based on network pharmacology. The specific chemical compounds of the essential oil were identified by GC-MS/MS, so the prediction accuracy is relatively high. The bioinformatics and computational analyses in network pharmacology indicated that the essential oil in flowers of *Citrus grandis* ‘Tomentosa’ had the potential effects of treating pain, tumors, neuropsychiatric diseases, ophthalmic diseases, and respiratory system diseases through multiple pathways and targets. Among them, the most worthy of further research is α-selinene, targeting SLC6A2 to treat pain and neuropsychiatric disorders, and β-caryophyllene, to treat breast cancer and colon cancer.

The results will provide direct and reliable evidence for the wider research and application of *Citrus grandis* ‘Tomentosa’, which will help reduce resource waste and bring economic benefits.

## Figures and Tables

**Table 1:** Major essential oil components of flowers from *Citrus grandis* ‘Tomentosa’.

No.	RT (min)	Compound	Content (%)	CAS	SMILES
1	5.390	(1R)-(+)-α-Pinene	0.44	7785-70-8	CC1=CC[C@@H]2C[C@H]1C2(C)C
2	6.496	(−)-β-Pinene	1.36	18172-67-3	CC1([C@H]2CCC(=C)[C@@H]1C2)C
3	6.923	Myrcene	8.93	123-35-3	CC(=CCCC(=C)C=C)C
4	7.821	M-Cymene	2.75	535-77-3	CC1=CC(=CC=C1)C(C)C
5	7.942	(+)-Dipentene	3	5989-27-5	CC1=CC[C@@H](CC1)C(=C)C
6	8.508	1,3,6-Octatriene,3,7-dimethyl-	1.82	13877-91-3	CC(=CCC=C(C)C=C)C
7	8.850	γ-Terpinene	9.23	99-85-4	CC1=CCC(=CC1)C(C)C
8	9.201	2-Furanmethanol,5-ethenyltetrahydro-a,a,5-trimethyl-, (2R,5S)-rel-	1.41	5989-33-3	C[C@]1(CC[C@@H](O1)C(C)(C)O)C=C
9	9.655	Cyclohexene,3-methyl-6-(1-methylethylidene)-	1.42	586-63-0	CC1CCC(=C(C)C)C=C1
10	10.034	3,7-Dimethylocta-1,6-dien-3-ol	5.35	78-70-6	CC(=CCCC(C)(C=C)O)C
11	12.213	Terpinen-4-ol	0.39	562-74-3	CC1=CCC(CC1)(C(C)C)O
12	12.601	α-Terpineol	1.3	98-55-5	CC1=CCC(CC1)C(C)(C)O
13	13.310	2-(4-Methyl-cyclohex-3-enyl)-propionaldehyde	0.33	29548-14-9	CC1=CCC(CC1)C(C)C=O
14	13.695	Nerol	0.81	106-25-2	CC(=CCC/C(=C\CO)/C)C
15	14.587	Geraniol	1.68	106-24-1	CC(=CCC/C(=C/CO)/C)C
16	17.699	Methyl anthranilate	3.23	134-20-3	COC(=O)C1=CC=CC=C1N
17	20.462	β-Caryophyllene	11.42	87-44-5	C/C/1=C\CCC(=C)[C@H]2CC([C@@H]2CC1)(C)C
18	21.516	α-Caryophyllene	1.92	6753-98-6	C/C/1=C\CC(/C=C/C/C(=C/CC1)/C)(C)C
19	22.464	(−)-Germacrene D	7.4	23986-74-5	C/C/1=C\CCC(=C)/C=C/[C@@H](CC1)C(C)C
20	22.883	α-Selinene	1.06	473-13-2	CC1=CCC[C@]2([C@H]1C[C@@H](CC2)C(=C)C)C
21	22.924	(+)-Bicyclogermacrene	2.03	24703-35-3	CC1=CCC2C(C1)CC(CC=C2C)(C)C
22	23.045	α-Muurolene	0.75	31983-22-9	CC1=C[C@@H]2[C@H](CC1)C(=CC[C@H]2C(C)C)C
23	23.424	1H-Benzocyclohepten-7-ol,2,3,4,4a,5,6,7,8-octahydro-1,1,4a,7-tetramethyl-, (4aS,7S)-	0.94	6892-80-4	C[C@@]12CCCC(C1=CC[C@@](CC2)(C)O)(C)C
24	23.763	Naphthalene, 1,2,3,5,6,8a-hexahydro-4,7-dimethyl-1-(1-methylethyl)-	1.38	16729-01-4	CC1=CC2C(CCC(=C2CC1)C)C(C)C
25	24.748	Germacrene B	0.64	15423-57-1	C/C/1=C\CC/C(=C/CC(=C(C)C)CC1)/C
26	25.124	Nerolidol	14.8	40716-66-3	CC(=CCC/C(=C/CCC(C)(C=C)O)/C)C
27	25.416	Spathulenol	1.19	6750-60-3	C[C@@]1(CC[C@@H]2[C@@H]1[C@H]3[C@H](C3(C)C)CCC2=C)O
28	25.581	Caryophyllene oxide	1.39	1139-30-6	C[C@@]12CC[C@@H]3[C@H](CC3(C)C)C(=C)CC[C@H]1O2
29	27.157	Isospathulenol	0.61	88395-46-4	CC1=C2CC[C@]([C@H]2[C@H]3[C@H](C3(C)C)CC1)(C)O
30	27.660	(−)-tau-Muurolol	0.6	19912-62-0	CC1=C[C@H]2[C@@H](CC[C@]([C@H]2CC1)(C)O)C(C)C
31	28.145	α-Cadinol	0.8	481-34-5	CC1=C[C@H]2[C@@H](CC[C@@]([C@@H]2CC1)(C)O)C(C)C
32	29.028	4(15),5,10(14)-Germacratrien-1-ol	0.17	81968-62-9	CC(C)[C@@H]/1CCC(=C)C(CCC(=C)/C=C1)O
33	29.342	Cis-Trans-Farnesol	0.27	3790-71-4	CC(=CCC/C(=C/CC/C(=C\CO)/C)/C)C
34	29.796	Farnesol	5.52	4602-84-0	CC(=CCC/C(=C/CC/C(=C/CO)/C)/C)C
35	31.956	Methyl hexadecanoate	0.44	112-39-0	CCCCCCCCCCCCCCCC(=O)OC
36	32.183	5-(5-Methyl-1-methylen-4-hexenyl)-1-(4-methyl-3-pentenyl)-1-cyclohexen	0.6	20016-73-3	CC(=CCCC1=CCCC(C1)C(=C)CCC=C(C)C)C
37	32.248	Dibutyl phthalate	0.44	84-74-2	CCCCOC(=O)C1=CC=CC=C1C(=O)OCCCC
38	32.428	4-(6-methylhepta-1,5-dien-2-yl)-1-(4-methylpent-3-enyl)cyclohexene	0.35	20016-72-2	CC(=CCCC1=CCC(CC1)C(=C)CCC=C(C)C)C
39	33.134	Methyl linoleate	0.34	112-63-0	CCCCC/C=C\C/C=C\CCCCCCCC(=O)OC
40	33.184	Methyl linolenate	0.31	301-00-8	CC/C=C\C/C=C\C/C=C\CCCCCCCC(=O)OC
41	33.230	4,8,13-Cyclotetradecatriene-1,3-diol,1,5,9-trimethyl-12-(1-methylethyl)-	0.33	7220-78-2	C/C/1=C\CC/C(=C/C(CC(/C=C\C(CC1)C(C)C)(C)O)O)/C
42	33.612	4,7,10,13,16,19-Docosahexaenoicacid, (4Z,7Z,10Z,13Z,16Z,19Z)-	0.19	6217-54-5	CC/C=C\C/C=C\C/C=C\C/C=C\C/C=C\C/C=C\CCC(=O)O
43	33.653	Z-9-octadecyl-18-lactone	0.69	88395-46-4	CC1=C2CC[C@]([C@H]2[C@H]3[C@H](C3(C)C)CC1)(C)O

RT: Retention time.

**Table 2:** The major targets of essential oil components in flowers from *Citrus grandis* ‘Tomentosa’ and its relevant topological parameters.

Uniprot ID	Protein Name	Gene Name	Degree	Betweenness Centrality	Closeness Centrality	Average Shortest Path Length
Q07869	Peroxisome proliferator-activated receptor alpha	PPARA	26	0.033301	0.4511	2.216802
P14867	Gamma-aminobutyric acid receptor subunit alpha-1	GABRA1	25	0.020241	0.407735	2.452575
P35354	Prostaglandin G/H synthase 2	PTGS2	22	0.024147	0.431075	2.319783
P23975	Sodium-dependent noradrenaline transporter	SLC6A2	21	0.017634	0.403279	2.479675
P08172	Muscarinic acetylcholine receptor M2	CHRM2	19	0.005435	0.329759	3.03252
P11229	Muscarinic acetylcholine receptor M1	CHRM1	19	0.009314	0.366071	2.731707
P34972	Cannabinoid receptor 2	CNR2	19	0.012984	0.411371	2.430894
P23219	Prostaglandin G/H synthase 1	PTGS1	19	0.024813	0.43007	2.325203
P20309	Muscarinic acetylcholine receptor M3	CHRM3	17	0.008508	0.358252	2.791328
P10275	Androgen receptor	AR	17	0.013655	0.417421	2.395664
P11511	Aromatase	CYP19A1	16	0.00618	0.366071	2.731707
Q8NER1	Transient receptor potential cation channel subfamily V member 1	TRPV1	14	0.005349	0.356522	2.804878
P28845	Corticosteroid 11-beta-dehydrogenase isozyme 1	HSD11B1	13	0.005774	0.359299	2.783198
Q01959	Sodium-dependent dopamine transporter	SLC6A3	13	0.007051	0.361057	2.769648
P04035	3-hydroxy-3-methylglutaryl-coenzyme A reductase	HMGCR	13	0.008209	0.376915	2.653117
P06401	Progesterone receptor	PGR	13	0.011462	0.380805	2.626016
O00519	Fatty-acid amide hydrolase 1	FAAH	13	0.013109	0.417894	2.392954
P18031	Tyrosine-protein phosphatase non-receptor type 1	PTPN1	12	0.004097	0.357905	2.794038
P22303	Acetylcholinesterase	ACHE	11	0.004405	0.322835	3.097561
P19793	Retinoic acid receptor RXR-alpha	RXRA	11	0.008531	0.412752	2.422764

**Table 3:** Diseases obtained from TTD based on the major targets.

Target	Diseases	Target	Diseases
PPARA	High cholesterol level; hyperlipoproteinemia	CYP19A1	Hormonally-responsive breast cancer; Cushing disease
GABRA1	Respiratory distress syndrome; intractable insomnia	TRPV1	Knee osteoarthritis; Atopic dermatitis
PTGS2	Chronic pain; musculoskeletal pain	HSD11B1	Influenza virus infection; lupus
SLC6A2	Acute pain; attention deficit hyperactivity disorder	SLC6A3	Attention deficit hyperactivity disorder; anesthesia
CHRM2	Asthma; glaucoma/ocular hypertension	HMGCR	Dyslipidemia; multiple sclerosis
CHRM1	Visceral spasms; anesthesia	PGR	Menstrual disorder; premature labor
CNR2	Attention deficit hyperactivity disorder; insomnia	FAAH	anesthesia; major depressive disorder
PTGS1	Miosis; dysmenorrhea	PTPN1	Ophthalmic surgery injury; infectious disease
CHRM3	Asthma; obstructive lung disease	ACHE	Open-angle glaucoma; Glaucoma/ocular hypertension
AR	Acute myeloid leukemia; alcoholic hepatitis	RXRA	Kaposi sarcoma; cutaneous T-cell lymphoma

## Data Availability

All data generated or analyzed during this study are included in this published article [and its Supplementary Information Files].

## Supplementary Materials

The following supporting information can be downloaded at https://www.mdpi.com/article/10.3390/cimb47070541/s1.

## References

[1] Kong F.S., Ding Z.D., Zhang K., Duan W.J., Qin Y.R., Su Z.P., Bi Y.G. (2020). Optimization of extraction flavonoids from and evaluation its hypoglycemic and hypolipidemic activities. J. Ethnopharmacol..

[2] Zeng X., Su W.W., Zheng Y.Y., Liu H., Li P.L., Zhang W.J., Liang Y.T., Bai Y., Peng W., Yao H.L. (2018). UFLC-Q-TOF-MS/MS-Based Screening and Identification of Flavonoids and Derived Metabolites in Human Urine after Oral Administration of Exocarpium Citri Grandis Extract. Molecules.

[3] Yu X.X., Liu Q.D., Wu J.W., Liang Z.K., Zhao M.Q., Xu X.J. (2016). Simultaneous Determination of Four Major Constituents in Citri Grandis Exocarpium by HPLC-DAD. Acta Chromatogr..

[4] Xie Z.S., Liu Q.D., Liang Z.K., Zhao M.Q., Yu X.X., Yang D.P., Xu X.J. (2013). The GC/MS Analysis of Volatile Components Extracted by Different Methods from Exocarpium Citri Grandis. J. Anal. Methods Chem..

[5] Su Z.P., Qin Y.R., Zhang K., Bi Y.G., Kong F.S. (2019). Inclusion Complex of Exocarpium Citri Grandis Essential Oil with β-Cyclodextrin: Characterization, Stability, and Antioxidant Activity. J. Food Sci..

[6] You J.S., He S.C., Chen L., Guo Z.H., Gao F., Zhang M.Y., Dan L., Chen W. (2023). Analysis of Pharmacological Activities and Mechanisms of Essential Oil in Leaves of C. grandis ‘Tomentosa’ by GC-MS/MS and Network Pharmacology. Comb. Chem. High Throughput Screen..

[7] Qi Y., Chen Q.Z., Oouyang Q.Q., Wang H.R., Chen X.B. (2022). Comparison of volatile oil composition between flowers and fruits of Exocarpium Citri Grandis. J. Guangdong Med. Univ..

[8] Paradis D., Bérail G., Bonmatin J.M., Belzunces L.P. (2014). Sensitive analytical methods for 22 relevant insecticides of 3 chemical families in honey by GC-MS/MS and LC-MS/MS. Anal. Bioanal. Chem..

[9] Hopkins A.L. (2008). Network pharmacology: The next paradigm in drug discovery. Nat. Chem. Biol..

[10] Zhou Z.C., Chen B., Chen S.M., Lin M.Q., Chen Y., Jin S., Chen W.Y., Zhang Y.Y. (2020). Applications of Network Pharmacology in Traditional Chinese Medicine Research. Evid.-Based Complement. Altern. Med..

[11] Luo T.T., Lu Y., Yan S.K., Xiao X., Rong X.L., Guo J. (2020). Network Pharmacology in Research of Chinese Medicine Formula: Methodology, Application and Prospective. Chin. J. Integr. Med..

[12] Peng J.J., Lu G.L., Xue H.S., Wang T., Shang X.Q. (2019). TS-GOEA: A web tool for tissue-specific gene set enrichment analysis based on gene ontology. BMC Bioinform..

[13] Liang B., Li C.N., Zhao J.Y. (2016). Identification of key pathways and genes in colorectal cancer using bioinformatics analysis. Med. Oncol..

[14] Zhou Y., Zhang Y.T., Zhao D.H., Yu X.Y., Shen X.Y., Zhou Y., Wang S.S., Qiu Y.Q., Chen Y.Z., Zhu F. (2024). TTD: Therapeutic Target Database describing target druggability information. Nucleic Acids Res..

[15] Simstein R., Burow M., Parker A., Weldon C., Beckman B. (2003). Apoptosis, chemoresistance, and breast cancer: Insights from the MCF-7 cell model system. Exp. Biol. Med..

[16] Kim N.D., Im E., Yoo Y.H., Choi Y.H. (2006). Modulation of the cell cycle and induction of apoptosis in human cancer cells by synthetic bile acids. Curr. Cancer Drug Targets.

[17] Ramalho T.R.D., de Oliveira M.T.P., Lima A.L.D., Bezerra-Santos C.R., Piuvezam M.R. (2015). Gamma-Terpinene Modulates Acute Inflammatory Response in Mice. Planta Med..

[18] Cetin H., Cilek J.E., Oz E., Aydin L., Deveci O., Yanikoglu A. (2010). Acaricidal activity of L. essential oil and its major components, carvacrol and γ-terpinene against adult (Acari: Ixodidae). Vet. Parasitol..

[19] Sousa-Pimenta M., Estevinho L.M., Szopa A., Basit M., Khan K., Armaghan M., Ibrayeva M., Gürer E.S., Calina D., Hano C. (2023). Chemotherapeutic properties and side-effects associated with the clinical practice of terpene alkaloids: Paclitaxel, docetaxel, and cabazitaxel. Front. Pharmacol..

[20] Winnacker M. (2023). Polyamides Derived from Terpenes: Advances in Their Synthesis, Characterization and Applications. Eur. J. Lipid Sci. Tech..

[21] Dahham S.S., Tabana Y., Asif M., Ahmed M., Babu D., Hassan L.E., Ahamed M.B.K., Sandai D., Barakat K., Siraki A. (2021). β-Caryophyllene Induces Apoptosis and Inhibits Angiogenesis in Colorectal Cancer Models. Int. J. Mol. Sci..

[22] Wang G.F., Ma W.B., Du J.W. (2018). β-Caryophyllene (BCP) ameliorates MPP plus induced cytotoxicity. Biomed. Pharmacother..

[23] Fidyt K., Fiedorowicz A., Strzadala L., Szumny A. (2016). β-caryophyllene and β-caryophyllene oxide-natural compounds of anticancer and analgesic properties. Cancer Med..

[24] Legault J., Pichette A. (2007). Potentiating effect of β-caryophyllene on anticancer activity of α-humulene, isocaryophyllene and paclitaxel. J. Pharm. Pharmacol..

[25] Kane W.J., Hassinger T.E., Elwood N.R., Dietch Z.C., Krebs E.D., Popovsky K.A., Hedrick T.L., Sawyer R.G. (2021). Fever Is Associated with Reduced Mortality in Trauma and Surgical Intensive Care Unit-Acquired Infections. Surg. Infect..

[26] Ceneviva G.D., Phipps L.M., Harter A.J., Mauger D.T., Lucking S.E., Thomas N.J., Dettorre M.D., Cilley R.E. (2001). Fever as a predictor of infection in childhood trauma. Crit. Care Med..

[27] ‘Sullivan D.O., Stanczak M.A., Villa M., Uhl F.M., Corrado M., Geltink R.I.K., Sanin D.E., Apostolova P., Rana N., Edwards-Hicks J. (2021). Fever supports CD8+effector T cell responses by mitochondrial translation. Proc. Natl. Acad. Sci. USA.

[28] Mier J.W., Souza L.M., Allegretta M., Boone T., Bernheim H.A., Dinarello C.A. (1985). Dissimilarities Between Purified Human Interleukin-1 and Recombinant Human Interleukin-2 in the Induction of Fever, Brain Prostaglandin, and Acute-Phase Protein-Synthesis. J. Biol. Response Modif..

[29] Seo Y., Prome S.A., Kim L., Han J.Y., Kim J.M., Choi S.J. (2022). Florid lambda-monotypic B-cell proliferation in fatal severe fever with thrombocytopenia syndrome virus infection-associated necrotizing lymphadenitis: A potential diagnostic pitfall. J. Hematop..

[30] Wrotek S., Sobocinska J., Kozlowski H.M., Pawlikowska M., Jedrzejewski T., Dzialuk A. (2020). New Insights into the Role of Glutathione in the Mechanism of Fever. Int. J. Mol. Sci..

[31] Kleef R., Jonas W.B., Knogler W., Stenzinger W. (2001). Fever, cancer incidence and spontaneous remissions. Neuroimmunomodulation.

[32] Gregório H., Magalhaes T.R., Pires I., Prada J., Carvalho M.I., Queiroga F.L. (2021). The role of COX expression in the prognostication of overall survival of canine and feline cancer: A systematic review. Vet. Med. Sci..

[33] Consalvi S., Biava M., Poce G. (2015). COX inhibitors: A patent review (2011–2014). Expert. Opin. Ther. Pat..

[34] Pereira R.D. (2009). Selective Cyclooxygenase-2 (COX-2) Inhibitors Used for Preventing or Regressing Cancer. Recent Pat. Anti-Cancer.

[35] Etain B., Mathieu F., Jamain S., Henry C., Roy I., Bellivier F., Leboyer M. (2006). Norepinephrine transporter gene (SLC6A2) polymorphisms influence phenotypic expression of bipolar disorder. Biol. Psychiatry.

[36] Fichna J.P., Huminska-Lisowska K., Safranow K., Adamczyk J.G., Cieszczyk P., Zekanowski C., Berdynski M. (2021). Rare Variant in the SLC6A2 Encoding a Norepinephrine Transporter Is Associated with Elite Athletic Performance in the Polish Population. Genes.

[37] Licinio J. (2010). Sequence Variability in ABCB1, SLC6A2, SLC6A3, SLC6A4, CREB1, CRHR1, NTRK2 and BDNF: Novel Variations and Association with Depression and Antidepressant Response. Biol. Psychiatry.

[38] Ono K., Iwanaga Y., Mannami T., Kokubo Y., Tomoike H., Komamura K., Shioji K., Yasui N., Tago N., Iwai N. (2003). Epidemiological evidence of an association between gene polymorphism and hypertension. Hypertens. Res..

[39] Singh A.K., Chanotiya C.S., Yadav A., Kalra A. (2010). Volatiles of: A Rich Source of Selinene Isomers. Nat. Prod. Commun..

