# Supplementary material for: Analysis of Pharmacological Activities and Mechanisms of Essential Oil in Flowers of Citrus grandis ‘Tomentosa’ by GC-MS/MS and Network Pharmacology
Source: Curr Issues Mol Biol. 2025 Jul 11;47(7):541. doi: 10.3390/cimb47070541 (PMC12293301; doi:10.3390/cimb47070541)
Supplement: Supplementary file 1 [file cimb-47-00541-s001.zip › cimb-3682589-supplementary.pdf]

Table S1 Information on potential targets of the essential oil composition in flowers of *C. grandis* ‘Tomentosa’

Ingredients	Targets	Uniprot ID	Description
1	GABRA2	P47869	Gamma-aminobutyric-acid receptor alpha-2 subunit
1	GABRA5	P31644	Gamma-aminobutyric-acid receptor alpha-5 subunit
1	GABRA1	P14867	Gamma-aminobutyric acid receptor subunit alpha-1
1	GABRA6	Q16445	Gamma-aminobutyric-acid receptor subunit alpha-6
1	PTGS2	P35354	Prostaglandin G/H synthase 2
1	PRKACA	P17612	mRNA of PKA Catalytic Subunit C-alpha
1	CHRNA2	Q15822	Neuronal acetylcholine receptor subunit alpha-2
1	PPARA	Q07869	Peroxisome proliferator-activated receptor alpha
1	CNR2	P34972	Cannabinoid receptor 2
1	ACHE	P22303	Acetylcholinesterase
1	FAAH	O00519	Anandamide amidohydrolase
1	TRPV1	Q8NER1	Vanilloid receptor
1	AR	P10275	Androgen Receptor (by homology)
1	CYP19A1	P11511	Cytochrome P450 19A1
1	ESR1	P03372	Estrogen receptor alpha
1	CHRM2	P08172	Muscarinic acetylcholine receptor M2
1	SLC6A2	P23975	Norepinephrine transporter
1	SLC6A4	P31645	Serotonin transporter
1	CYP2C19	P33261	Cytochrome P450 2C19
1	BCHE	P06276	Butyrylcholinesterase
1	PTPN1	P18031	Protein-tyrosine phosphatase 1B
1	CXCR3	P49682	C-X-C chemokine receptor type 3
1	TDP1	Q9NUW8	Tyrosyl-DNA phosphodiesterase 1
2	GABRA2	P47869	Gamma-aminobutyric-acid receptor alpha-2 subunit
2	GABRA1	P14867	Gamma-aminobutyric acid receptor subunit alpha-1
2	PTGS1	P23219	Prostaglandin G/H synthase 1
2	PTGS2	P35354	Prostaglandin G/H synthase 2
2	CHRM3	P20309	Muscarinic acetylcholine receptor M3
2	CHRM1	P11229	Muscarinic acetylcholine receptor M1
2	RXRA	P19793	Retinoic acid receptor RXR-alpha
2	SLC6A2	P23975	Sodium-dependent noradrenaline transporter

2	GABRA3	P34903	Gamma-aminobutyric-acid receptor alpha-3 subunit
2	CHRM2	P08172	Muscarinic acetylcholine receptor M2
2	ADRA1B	P35368	Alpha-1B adrenergic receptor
2	NCOA2	Q15596	Nuclear receptor coactivator 2
2	GABRA6	Q16445	Gamma-aminobutyric-acid receptor subunit alpha-6
2	ADRA2C	P18825	Alpha-2C adrenergic receptor
2	ADRA1A	P35348	Alpha-1A adrenergic receptor
2	SLC6A3	Q01959	Sodium-dependent dopamine transporter
2	MAOA	P21397	Amine oxidase [flavin-containing] A
2	GABRA5	P31644	Gamma-aminobutyric-acid receptor alpha-5 subunit
2	CHRNA2	Q15822	Neuronal acetylcholine receptor subunit alpha-2
3	ADH1C	P00326	Alcohol dehydrogenase 1C
3	GABRA1	P14867	Gamma-aminobutyric acid receptor subunit alpha-1
3	SQLE	Q14534	Squalene monooxygenase
3	GGPS1	O95749	Geranylgeranyl pyrophosphate synthase
3	FNTB	P49356	Protein farnesyltransferase subunit beta
3	PPARA	Q07869	Peroxisome proliferator-activated receptor alpha
3	CNR2	P34972	Cannabinoid receptor 2
4	SLC6A2	P23975	Sodium-dependent noradrenaline transporter
4	RNASEH1	O60930	Ribonuclease H1
4	TYR	P14679	Tyrosinase
4	KCNK9	Q9NPC2	Potassium channel subfamily K member 9
4	GABRB1	P18505	Gamma-aminobutyric acid receptor subunit beta-1
4	CHRNG	P07510	Acetylcholine receptor subunit gamma
4	ENPP3	O14638	Ectonucleotide pyrophosphatase/phosphodiesterase family member 3
4	CHRNB1	P11230	Acetylcholine receptor subunit beta
4	CHRND	Q07001	Acetylcholine receptor subunit delta
4	COPS5	Q92905	COP9 signalosome complex subunit 5
4	PFKFB4	Q16844	6-phosphofructo-2-kinase/fructose-2,6-bisphosphatase 4
4	CHRNA1	P02708	Acetylcholine receptor subunit alpha
4	CTBP2	P[56545	C-terminal-binding protein 2
4	SCN4A	P35499	Sodium channel protein type 4 subunit alpha
4	ALB	P02768	Serum albumin

4	PFKFB3	Q16875	6-phosphofructo-2-kinase/fructose-2,6-bisphosphatase 3
4	GPR139	Q6DWJ6	Probable G-protein coupled receptor 139
4	GABRA1	P14867	Gamma-aminobutyric acid receptor subunit alpha-1
4	CRBRB2	P47870	Gamma-aminobutyric acid receptor subunit beta-2
4	GRBRG2	P18507	Gamma-aminobutyric acid receptor subunit gamma-2
4	PPARA	Q07869	Peroxisome proliferator-activated receptor alpha
4	HTR2B	P41595	Family A G protein-coupled receptor
4	CA2	P00918	Lyase
4	GRBRB3	P28472	Gamma-aminobutyric acid receptor subunit beta-3
4	PTGS1	P23219	Prostaglandin G/H synthase 1
4	HTR2C	P28335	Family A G protein-coupled receptor
4	ACHE	P22303	Acetylcholinesterase
4	TAAR1	Q96RJ0	Family A G protein-coupled receptor
5	PTGS2	P35354	Prostaglandin G/H synthase 2
5	GABRA1	P14867	Gamma-aminobutyric acid receptor subunit alpha-1
5	ADH1C	P00326	Alcohol dehydrogenase 1C
5	ADH1B	P00325	Alcohol dehydrogenase 1B
5	NCOA2	Q15596	Nuclear receptor coactivator 2
5	NCOA1	Q15788	Nuclear receptor coactivator 1
5	CHRM2	P08172	Muscarinic acetylcholine receptor M2
5	GABRA2	P47869	Gamma-aminobutyric-acid receptor alpha-2 subunit
5	CHRM1	P11229	Muscarinic acetylcholine receptor M1
5	GABRA5	P31644	Gamma-aminobutyric-acid receptor alpha-5 subunit
5	GABRA6	Q16445	Gamma-aminobutyric-acid receptor subunit alpha-6
5	GABRA3	P34903	Gamma-aminobutyric-acid receptor alpha-3 subunit
5	IGHG1	P01857	Ig gamma-1 chain C region
5	PPARG	P37231	Peroxisome proliferator-activated receptor gamma
5	CYP2C9	P11712	Cytochrome P450 2C9
5	CYP2C19	P33261	Cytochrome P450 2C19
5	PPARA	Q07869	Peroxisome proliferator-activated receptor alpha

5	CNR2	P34972	Cannabinoid receptor 2
5	CYP19A1	P11511	Cytochrome P450 19A1
6	PPARA	Q07869	Peroxisome proliferator-activated receptor alpha
6	CNR2	P34972	Cannabinoid receptor 2
7	PTGS2	P35354	Prostaglandin G/H synthase 2
7	ACHE	P22303	Acetylcholinesterase
7	GABRA1	P14867	Gamma-aminobutyric acid receptor subunit alpha-1
7	DPP4	P27487	Dipeptidyl peptidase IV
7	ADH1C	P00326	Alcohol dehydrogenase 1C
7	ADH1B	P00325	Alcohol dehydrogenase 1B
7	ADH1A	P07327	Alcohol dehydrogenase 1A
7	TRPV1	Q8NER1	Vanilloid receptor
7	PLK1	P53350	Serine/threonine-protein kinase PLK1
7	GLI2	P10070	Zinc finger protein GLI2
7	GLI1	P08151	Zinc finger protein GLI1
8	CHRM3	P20309	Muscarinic acetylcholine receptor M3
8	CHRM1	P11229	Muscarinic acetylcholine receptor M1
8	GABRA5	P31644	Gamma-aminobutyric-acid receptor alpha-5 subunit
8	GABRA3	P34903	Gamma-aminobutyric-acid receptor alpha-3 subunit
8	GABRA2	P47869	Gamma-aminobutyric-acid receptor alpha-2 subunit
8	CHRM2	P08172	Muscarinic acetylcholine receptor M2
8	ADRA1B	P35368	Alpha-1B adrenergic receptor
8	CHRNA2	Q15822	Neuronal acetylcholine receptor subunit alpha-2
8	GABRA1	P14867	Gamma-aminobutyric acid receptor subunit alpha-1
8	IGHG1	P01857	Ig gamma-1 chain C region
8	IGHG2	P01859	Ig gamma-2 chain C region
8	GABRA6	Q16445	Gamma-aminobutyric-acid receptor subunit alpha-6
8	GABRA4	P48169	Gamma-aminobutyric-acid receptor subunit alpha-4
8	HSD17B2	P37059	Estradiol 17-beta-dehydrogenase 2
8	CYP11B1	P15538	Cytochrome P450 11B1
8	CYP11B2	P19099	Cytochrome P450 11B2
8	LRRK2	Q5S007	Leucine-rich repeat serine/threonine-protein kinase 2
8	FKBP1A	P62942	FK506-binding protein 1A

8	AR	P10275	Androgen Receptor
8	NR3C1	P04150	Glucocorticoid receptor
8	PGR	P06401	Progesterone receptor
8	CDK5R1	Q15078	Cyclin-dependent kinase 5 activator 1
8	CDK5	Q00535	Cyclin-dependent-like kinase 5
8	BRD4	O60885	Bromodomain-containing protein 4
8	BRD2	P25440	Bromodomain-containing protein 2
8	GSR	P00390	Glutathione reductase
8	AOC3	Q16853	Amine oxidase, copper containing
8	CYP51A1	Q16850	Cytochrome P450 51
8	PTAFR	P25105	Platelet activating factor receptor
8	DRD4	P21917	Dopamine D4 receptor
8	FAAH	O00519	Anandamide amidohydrolase
8	CASP1	P29466	Caspase-1
8	JAK3	P52333	Tyrosine-protein kinase JAK3
8	JAK1	P23458	Tyrosine-protein kinase JAK1
8	JAK2	O60674	Tyrosine-protein kinase JAK2
8	TYK2	P29597	Tyrosine-protein kinase TYK2
9	GABRA1	P14867	Gamma-aminobutyric acid receptor subunit alpha-1
9	AR	P10275	Androgen Receptor
9	CYP19A1	P11511	Cytochrome P450 19A1
9	ESR1	P03372	Estrogen receptor alpha
9	ESR2	Q92731	Estrogen receptor beta
9	RBP4	P02753	Plasma retinol-binding protein
9	PPARA	Q07869	Peroxisome proliferator-activated receptor alpha
9	CNR2	P34972	Cannabinoid receptor 2
10	CHRM3	P20309	Muscarinic acetylcholine receptor M3
10	CHRM1	P11229	Muscarinic acetylcholine receptor M1
10	CHRM2	P08172	Muscarinic acetylcholine receptor M2
10	GABRA1	P14867	Gamma-aminobutyric acid receptor subunit alpha-1
10	GABRA5	P31644	Gamma-aminobutyric-acid receptor alpha-5 subunit
10	GABRA3	P34903	Gamma-aminobutyric-acid receptor alpha-3 subunit
10	GABRA2	P47869	Gamma-aminobutyric-acid receptor alpha-2 subunit
10	GABRA6	Q16445	Gamma-aminobutyric-acid receptor subunit alpha-6
10	MTRR	Q9UBK8	Methionine synthase reductase
10	POR	P16435	NADPH--cytochrome P450 reductase

10	TRPA1	O75762	Transient receptor potential cation channel subfamily A member 1
10	NOS1	P29475	Nitric oxide synthase, brain
10	NOS2	P35228	Nitric oxide synthase, inducible
10	NOS3	P29474	Nitric oxide synthase, endothelial
10	TP53	P04637	Cellular tumor antigen p53
10	HMGCR	P04035	3-hydroxy-3-methylglutaryl-coenzyme A reductase
10	TRPV3	Q8NET8	Transient receptor potential cation channel subfamily V member 3
10	CA2	P00918	Carbonic anhydrase II
10	CA1	P00915	Carbonic anhydrase I
10	CA4	P22748	Carbonic anhydrase IV
10	TRPM8	Q7Z2W7	Transient receptor potential cation channel subfamily M member 8
10	NR3C2	P08235	Mineralocorticoid receptor
10	NR3C1	P04150	Glucocorticoid receptor
10	PGR	P06401	Progesterone receptor
10	SIGMAR1	Q99720	Sigma opioid receptor
10	SLC6A3	Q01959	Dopamine transporter (by homology)
10	SQLE	Q14534	Squalene monooxygenase
10	IDO1	P14902	Indoleamine 2,3-dioxygenase
10	HSD17B2	P37059	Estradiol 17-beta-dehydrogenase 2
10	DRD2	P14416	Dopamine D2 receptor (by homology)
10	CHRM4	P08173	Muscarinic acetylcholine receptor M4
10	OPRM1	P35372	Mu opioid receptor
10	OPRD1	P41143	Delta opioid receptor
10	OPRK1	P41145	Kappa Opioid receptor
10	ADRA2C	P18825	Adrenergic receptor alpha-2
10	HMOX1	P09601	Heme oxygenase 1 (by homology)
10	JAK1	P23458	Tyrosine-protein kinase JAK1
10	JAK2	O60674	Tyrosine-protein kinase JAK2
10	PTGS2	P35354	Cyclooxygenase-2
10	KCNA5	P22460	Voltage-gated potassium channel subunit Kv1.5
10	PTAFR	P25105	Platelet activating factor receptor
10	SCN5A	Q14524	Sodium channel protein type V alpha subunit
10	SCN9A	Q15858	Sodium channel protein type IX alpha subunit
10	PARP1	P09874	Poly [ADP-ribose] polymerase-1
10	ADRA1A	P35348	Alpha-1a adrenergic receptor
10	HRH3	Q9Y5N1	Histamine H3 receptor
10	HRH4	Q9H3N8	Histamine H4 receptor
10	JAK3	P52333	Tyrosine-protein kinase JAK3

10	TYK2	P29597	Tyrosine-protein kinase TYK2
10	TNNC1	P63316	Troponin C, slow skeletal and cardiac muscles
10	TNNT2	P45379	Troponin T, cardiac muscle
10	TNNI3	P19429	Troponin I, cardiac muscle
10	LRRK2	Q5S007	Leucine-rich repeat serine/threonine-protein kinase 2
10	AR	P10275	Androgen Receptor
10	GGPS1	O95749	Geranylgeranyl pyrophosphate synthase
11	GABRA1	P14867	Gamma-aminobutyric acid receptor subunit alpha-1
11	GABRA2	P47869	Gamma-aminobutyric-acid receptor alpha-2 subunit
11	AR	P10275	Androgen Receptor
11	CYP19A1	P11511	Cytochrome P450 19A1
11	CA2	P00918	Carbonic anhydrase II
11	CA1	P00915	Carbonic anhydrase I
11	CA4	P22748	Carbonic anhydrase IV
11	RORC	P51449	Nuclear receptor ROR-gamma
11	SREBF2	Q12772	Sterol regulatory element-binding protein 2
11	NPC1L1	Q9UHC9	Niemann-Pick C1-like protein 1
11	NR1H3	Q13133	LXR-alpha
11	HMGCR	P04035	HMG-CoA reductase
11	TRPM8	Q7Z2W7	Transient receptor potential cation channel subfamily M member 8
11	ESR2	Q92731	Estrogen receptor beta
11	CHRM2	P08172	Muscarinic acetylcholine receptor M2
11	CYP17A1	P05093	Cytochrome P450 17A1
11	PTPN1	P18031	Protein-tyrosine phosphatase 1B
11	RORA	P35398	Nuclear receptor ROR-alpha
11	SQLE	Q14534	Squalene monooxygenase
11	DRD2	P14416	Dopamine D2 receptor (by homology)
11	SLC6A4	P31645	Serotonin transporter
11	CYP51A1	Q16850	Cytochrome P450 51
11	PPARA	Q07869	Peroxisome proliferator-activated receptor alpha
11	PPARD	Q03181	Peroxisome proliferator-activated receptor delta
11	G6PD	P11413	Glucose-6-phosphate 1-dehydrogenase
11	ESR1	P03372	Estrogen receptor alpha
11	ACHE	P22303	Acetylcholinesterase
11	ADRA2C	P18825	Adrenergic receptor alpha-2
11	HSD17B2	P37059	Estradiol 17-beta-dehydrogenase 2
11	SHBG	P04278	Testis-specific androgen-binding protein

11	SLC6A2	P23975	Norepinephrine transporter
11	NR3C1	P04150	Glucocorticoid receptor
11	NR3C2	P08235	Mineralocorticoid receptor
11	BCHE	P06276	Butyrylcholinesterase
11	PTPRF	P10586	Receptor-type tyrosine-protein phosphatase F (LAR)
11	PTPN2	P17706	T-cell protein-tyrosine phosphatase
11	PLA2G1B	P04054	Phospholipase A2 group 1B
11	ACP1	P24666	Low molecular weight phosphotyrosine protein phosphatase
11	AKR1B10	O60218	Aldo-keto reductase family 1 member B10
11	GLI2	P10070	Zinc finger protein GL2
11	GLI1	P08151	Zinc finger protein GLI1
12	CHRM3	P20309	Muscarinic acetylcholine receptor M3
12	CHRM1	P11229	Muscarinic acetylcholine receptor M1
12	CHRM2	P08172	Muscarinic acetylcholine receptor M2
12	GABRA5	P31644	Gamma-aminobutyric-acid receptor alpha-5 subunit
12	GABRA3	P34903	Gamma-aminobutyric-acid receptor alpha-3 subunit
12	GABRA2	P47869	Gamma-aminobutyric-acid receptor alpha-2 subunit
12	SLC6A2	P23975	Sodium-dependent noradrenaline transporter
12	ADRA1A	P35348	Alpha-1A adrenergic receptor
12	ADRA1B	P35368	Alpha-1B adrenergic receptor
12	SLC6A3	Q01959	Sodium-dependent dopamine transporter
12	GABRA1	P14867	Gamma-aminobutyric acid receptor subunit alpha-1
12	IGHG1	P01857	Ig gamma-1 chain C region
12	MAOB	P27338	Amine oxidase [flavin-containing] B
12	MAOA	P21397	Amine oxidase [flavin-containing] A
12	GABRA6	Q16445	Gamma-aminobutyric-acid receptor subunit alpha-6
12	ADRB1	P08588	Beta-1 adrenergic receptor
12	RXRA	P19793	Retinoic acid receptor RXR-alpha
12	PDE3A	Q14432	CGMP-inhibited 3',5'-cyclic phosphodiesterase A
12	HTR2A	P28223	5-hydroxytryptamine 2A receptor
12	ADRB2	P07550	Beta-2 adrenergic receptor
12	SLC6A4	P31645	Sodium-dependent serotonin transporter
12	AR	P10275	Androgen Receptor
12	CYP19A1	P11511	Cytochrome P450 19A1
12	CA2	P00918	Carbonic anhydrase II

12	CA1	P00915	Carbonic anhydrase I
12	CA4	P22748	Carbonic anhydrase IV
12	TRPM8	Q7Z2W7	Transient receptor potential cation channel subfamily M member 8
12	SLC6A4	P31645	Serotonin transporter
12	NR1H3	Q13133	LXR-alpha
12	PTPN1	P18031	Protein-tyrosine phosphatase 1B
12	NR1I3	Q14994	Nuclear receptor subfamily 1 group I member 3 (by homology)
12	SREBF2	Q12772	Sterol regulatory element-binding protein 2
12	NPC1L1	Q9UHC9	Niemann-Pick C1-like protein 1
12	BCHE	P06276	Butyrylcholinesterase
12	ESR1	P03372	Estrogen receptor alpha
12	SQLE	Q14534	Squalene monooxygenase
12	ACHE	P22303	Acetylcholinesterase
12	CYP51A1	Q16850	Cytochrome P450 51
12	SLC6A2	P23975	Norepinephrine transporter
12	DRD2	P14416	Dopamine D2 receptor (by homology)
12	CYP17A1	P05093	Cytochrome P450 17A1
12	ESR2	Q92731	Estrogen receptor beta
12	CYP2C19	P33261	Cytochrome P450 2C19
12	NR3C2	P08235	Mineralocorticoid receptor
12	PTPRF	P10586	Receptor-type tyrosine-protein phosphatase F (LAR)
12	PTPN2	P17706	T-cell protein-tyrosine phosphatase
12	PLA2G1B	P04054	Phospholipase A2 group 1B
12	ACP1	P24666	Low molecular weight phosphotyrosine protein phosphatase
12	AKR1B10	O60218	Aldo-keto reductase family 1 member B10
12	SIGMAR1	Q99720	Sigma opioid receptor
12	NR3C1	P04150	Glucocorticoid receptor
12	TRPV3	Q8NET8	Transient receptor potential cation channel subfamily V member 3
12	ATP12A	P54707	Potassium-transporting ATPase alpha chain 2
12	PTPN6	P29350	Protein-tyrosine phosphatase 1C
12	SHBG	P04278	Testis-specific androgen-binding protein
12	FABP4	P15090	Fatty acid binding protein adipocyte
12	PPARA	Q07869	Peroxisome proliferator-activated receptor alpha
12	FABP3	P05413	Fatty acid binding protein muscle
12	FABP5	Q01469	Fatty acid binding protein epidermal
12	PPARD	Q03181	Peroxisome proliferator-activated receptor delta

12	FABP1	P07148	Fatty acid-binding protein, liver
12	RORA	P35398	Nuclear receptor ROR-alpha
12	HMOX1	P09601	Heme oxygenase 1 (by homology)
12	HMGCR	P04035	HMG-CoA reductase
12	CD81	P60033	CD81 antigen
12	PGR	P06401	Progesterone receptor
12	G6PD	P11413	Glucose-6-phosphate 1-dehydrogenase
12	SCD	O00767	Acyl-CoA desaturase
12	ADRA2C	P18825	Adrenergic receptor alpha-2
12	HSD11B1	P28845	11-beta-hydroxysteroid dehydrogenase 1
12	SLC6A3	Q01959	Dopamine transporter
13	CYP19A1	P11511	Cytochrome P450 19A1
13	SRD5A1	P18405	Steroid 5-alpha-reductase 1
13	FAAH	O00519	Anandamide amidohydrolase
13	SRD5A2	P31213	Steroid 5-alpha-reductase 2
13	CTSD	P07339	Cathepsin D
13	TRPV1	Q8NER1	Vanilloid receptor
13	SIGMAR1	Q99720	Sigma opioid receptor
13	SERPINA6	P08185	Corticosteroid binding globulin
13	SHBG	P04278	Testis-specific androgen-binding protein
13	NR1I2	O75469	Pregnane X receptor
13	FABP1	P07148	Fatty acid-binding protein, liver (by homology)
13	ADH1A	P07327	Alcohol dehydrogenase alpha chain
13	PTGS1	P23219	Cyclooxygenase-1
13	AR	P10275	Androgen Receptor
13	CYP17A1	P05093	Cytochrome P450 17A1
13	CES2	O00748	Carboxylesterase 2
13	PGR	P06401	Progesterone receptor
13	HSD17B3	P37058	Estradiol 17-beta-dehydrogenase 3
13	PTPN6	P29350	Protein-tyrosine phosphatase 1C
13	PARP1	P09874	Poly [ADP-ribose] polymerase-1
13	PPARA	Q07869	Peroxisome proliferator-activated receptor alpha
13	FABP5	Q01469	Fatty acid binding protein epidermal
13	PPARD	Q03181	Peroxisome proliferator-activated receptor delta
13	PTPN1	P18031	Protein-tyrosine phosphatase 1B
13	IMPDH2	P12268	Inosine-5'-monophosphate dehydrogenase 2
13	CYP2A6	P11509	Cytochrome P450 2A6
13	MAOB	P27338	Monoamine oxidase B
13	CES1	P23141	Acyl coenzyme A: cholesterol acyltransferase
13	SLC6A3	Q01959	Dopamine transporter

13	ADH4	P08319	Alcohol dehydrogenase class II
13	ADH1B	P00325	Alcohol dehydrogenase beta chain
13	ADH1C	P00326	Alcohol dehydrogenase gamma chain
13	SIRT2	Q8IXJ6	NAD-dependent deacetylase sirtuin 2
13	TYMS	P04818	Thymidylate synthase
13	HTR7	P34969	Serotonin 7 (5-HT7) receptor
13	NR3C2	P08235	Mineralocorticoid receptor
13	CA2	P00918	Carbonic anhydrase II
13	CA1	P00915	Carbonic anhydrase I
13	ACE	P12821	Angiotensin-converting enzyme (by homology)
13	NR1I3	Q14994	Nuclear receptor subfamily 1 group I member 3 (by homology)
13	TOP1	P11387	DNA topoisomerase I
13	P2RX7	Q99572	P2X purinoceptor 7
13	NPC1L1	Q9UHC9	Niemann-Pick C1-like protein 1
13	DRD2	P14416	Dopamine D2 receptor
13	DRD4	P21917	Dopamine D4 receptor
13	TRPA1	O75762	Transient receptor potential cation channel subfamily A member 1
13	NOS2	P35228	Nitric oxide synthase, inducible
13	NR3C1	P04150	Glucocorticoid receptor
13	MAPK3	P27361	MAP kinase ERK1
13	PRKCH	P24723	Protein kinase C eta
13	HSD11B2	P80365	11-beta-hydroxysteroid dehydrogenase 2
13	PTPN11	Q06124	Protein-tyrosine phosphatase 2C
13	AKR1B10	O60218	Aldo-keto reductase family 1 member B10
13	CHRM1	P11229	Muscarinic acetylcholine receptor M1 (by homology)
13	HSD11B1	P28845	11-beta-hydroxysteroid dehydrogenase 1
13	HMOX1	P09601	Heme oxygenase 1 (by homology)
13	ALOX5	P09917	Arachidonate 5-lipoxygenase
13	CTSK	P43235	Cathepsin K
13	RORC	P51449	Nuclear receptor ROR-gamma
13	POLB	P06746	DNA polymerase beta
13	PLA2G1B	P04054	Phospholipase A2 group 1B
13	ACP1	P24666	Low molecular weight phosphotyrosine protein phosphatase
13	G6PD	P11413	Glucose-6-phosphate 1-dehydrogenase
13	CYP51A1	Q16850	Cytochrome P450 51 (by homology)
13	DNMT3A	Q9Y6K1	DNA (cytosine-5)-methyltransferase 3A
13	KAT2B	Q92831	Histone acetyltransferase PCAF
13	SLC5A7	Q9GZV3	High-affinity choline transporter (by

			homology)
13	MAOA	P21397	Monoamine oxidase A
13	MMP13	P45452	Matrix metalloproteinase 13
13	MMP1	P03956	Matrix metalloproteinase 1
14	ADH1C	Q9Z4J7	Alcohol dehydrogenase 1C
14	TRPV1	Q8NER1	Transient receptor potential cation channel subfamily V member 1
14	TRPA1	O75762	Transient receptor potential cation channel subfamily A member 1
14	SQLE	Q14534	Squalene monooxygenase
14	PTGS1	P23219	Cyclooxygenase-1
14	PTGS2	P35354	Cyclooxygenase-2
14	HMGCR	P04035	HMG-CoA reductase (by homology)
14	PGR	P06401	Progesterone receptor
14	JAK1	P23458	Tyrosine-protein kinase JAK1
14	JAK2	O60674	Tyrosine-protein kinase JAK2
14	UGT2B7	P16662	UDP-glucuronosyltransferase 2B7
14	KCNH2	Q12809	HERG
14	CYP11B1	P15538	Cytochrome P450 11B1
14	CYP11B2	P19099	Cytochrome P450 11B2
14	IDO1	P14902	Indoleamine 2,3-dioxygenase
14	EPHX2	P34913	Epoxide hydratase
14	PIM1	P11309	Serine/threonine-protein kinase PIM1
14	HSD11B1	P28845	11-beta-hydroxysteroid dehydrogenase 1
14	PIM3	Q86V86	Serine/threonine-protein kinase PIM3
15	ADH1C	Q9Z4J7	Alcohol dehydrogenase 1C
15	ADH1B	P00325	Alcohol dehydrogenase 1B
15	PGR	P06401	Progesterone receptor
15	CCND1	P24385	G1/S-specific cyclin-D1
15	MAPK3	P27361	Mitogen-activated protein kinase 3
15	CDK4	P11802	Cell division protein kinase 4
15	BAK1	Q16611	Bcl-2 homologous antagonist/killer
15	PRKCB	P05771	Protein kinase C beta type
15	HMGCR	P04035	3-hydroxy-3-methylglutaryl-coenzyme A reductase
15	CYP2B6	P20813	Cytochrome P450 2B6
15	SI	P14410	Sucrase-isomaltase, intestinal
15	LCT	P09848	Lactase-phlorizin hydrolase
15	CDK2	P24941	Cyclin-dependent kinase 2
15	DDC	P20711	Aromatic-L-amino-acid decarboxylase
15	IPP	Q9Y573	Actin-binding protein IPP
15	GGPS1	O95749	Geranylgeranyl pyrophosphate synthase
15	LSS	P48449	Lanosterol synthase

15	BTN3A1	O00481	Butyrophilin subfamily 3 member A1
15	FNTB	P49356	Protein farnesyltransferase subunit beta
15	FNTA	P49354	Protein farnesyltransferase/ geranylgeranyltransferase type-1 subunit alpha
15	CDC25C	P30307	M-phase inducer phosphatase 3
15	SQLE	Q14534	Squalene monooxygenase
15	PTGS1	P23219	Cyclooxygenase-1
15	PTGS2	P35354	Cyclooxygenase-2
15	HMGCR	P04035	HMG-CoA reductase (by homology)
15	PGR	P06401	Progesterone receptor
15	JAK1	P23458	Tyrosine-protein kinase JAK1
15	JAK2	O60674	Tyrosine-protein kinase JAK2
15	UGT2B7	P16662	UDP-glucuronosyltransferase 2B7
15	KCNH2	Q12809	Potassium voltage-gated channel subfamily H member 2
15	CYP11B1	P15538	Cytochrome P450 11B1
15	CYP11B2	P19099	Cytochrome P450 11B2
15	IDO1	P14902	Indoleamine 2,3-dioxygenase
15	EPHX2	P34913	Epoxide hydratase
15	PIM1	P11309	Serine/threonine-protein kinase PIM1
15	HSD11B1	P28845	11-beta-hydroxysteroid dehydrogenase 1
15	PIM3	Q86V86	Serine/threonine-protein kinase PIM3
16	PTGS1	P23219	Prostaglandin G/H synthase 1
16	PTGS2	P35354	Prostaglandin G/H synthase 2
16	SLC6A2	P23975	Sodium-dependent noradrenaline transporter
16	SLC6A3	Q01959	Sodium-dependent dopamine transporter
16	MAOA	P21397	Amine oxidase [flavin-containing] A
16	ACR	P10323	Acrosin
16	AKR1C1	Q04828	Aldo-keto reductase family 1 member C1
16	AKR1C2	P52895	Aldo-keto reductase family 1 member C2
16	AKR1C3	P42330	Aldo-keto reductase family 1 member C3
16	PRMT5	O14744	Protein arginine N-methyltransferase 5
16	BDKRB1	P46663	B1 bradykinin receptor
16	CACNA1D	Q01668	Voltage-dependent L-type calcium channel subunit alpha-1D
16	CA7	P43166	Carbonic anhydrase 7
16	CYP1A2	P05177	Cytochrome P450 1A2
16	CYP2C9	P11712	Cytochrome P450 2C9
16	CYP2C19	P33261	Cytochrome P450 2C19
16	ESRRA	P11474	Steroid hormone receptor ERR1
16	CES1	P23141	Liver carboxylesterase 1
16	CES2	O00748	Cocaine esterase

16	F12	P00748	Coagulation factor XII
16	RCE1	Q9Y256	CAAX prenyl protease 2
16	FADS1	O60427	Acyl-CoA (8-3)-desaturase
16	FGB	P02675	Fibrinogen beta chain
16	FUT7	Q11130	Alpha-(1,3)-fucosyltransferase 7
16	GRK6	P43250	G protein-coupled receptor kinase 6
16	GRM6	O15303	Metabotropic glutamate receptor 6
16	GSTA1	P08263	Glutathione S-transferase A1
16	HDAC1	Q13547	Histone deacetylase 1
16	HDAC2	Q92769	Histone deacetylase 2
16	HDAC3	O15379	Histone deacetylase 3
16	HMGB1	P09429	High mobility group protein B1
16	EPHX1	P07099	Epoxide hydrolase 1
16	KCNMA1	Q12791	Calcium-activated potassium channel subunit alpha-1
16	KCNK9	Q9NPC2	Potassium channel subfamily K member 9
16	KDM3A	Q9Y4C1	Lysine-specific demethylase 3A
16	KYNU	Q16719	Kynureninase
16	LNPEP	Q9UIQ6	Leucyl-cystinyl aminopeptidase
16	LOXL2	Q9Y4K0	Lysyl oxidase homolog 2
16	WDR77	Q9BQA1	Methylosome protein 50
16	MAPK10	P53779	Mitogen-activated protein kinase 10
16	MMP16	P51512	Matrix metalloproteinase-16
16	NCOA1	Q15788	Nuclear receptor coactivator 1
16	NCOA3	Q9Y6Q9	Nuclear receptor coactivator 3
16	NLRP1	Q9C000	NACHT, LRR and PYD domains-containing protein 1
16	NNMT	P40261	Nicotinamide N-methyltransferase
16	NQO2	P16083	Ribosyldihydronicotinamide dehydrogenase [quinone]
16	PLA2G2A	P14555	Phospholipase A2, membrane associated
16	PARP10	Q53GL7	Protein mono-ADP-ribosyltransferase PARP10
16	PASK	Q96RG2	PAS domain-containing serine/threonine-protein kinase
16	PDK2	Q15119	[Pyruvate dehydrogenase (acetyl-transferring)] kinase isozyme 2, mitochondrial
16	ALPL	P05186	Alkaline phosphatase, tissue-nonspecific isozyme
16	PRTN3	P24158	Myeloblastin
16	SENP7	Q9BQF6	Sentrin-specific protease 7
16	SERPINH1	P50454	Serpin H1
16	SETD7	Q8WTS6	Histone-lysine N-methyltransferase SETD7

16	TRPA1	O75762	Transient receptor potential cation channel subfamily A member 1
16	TAS1R1	Q7RTX1	Taste receptor type 1 member 1
16	CA2	P00918	Carbonic anhydrase II
16	CA1	P00915	Carbonic anhydrase I
16	HTT	P42858	Huntingtin
16	TAAR1	Q96RJ0	Trace amine-associated receptor 1 (by homology)
16	KDR	P35968	Vascular endothelial growth factor receptor 2
16	ADRA2B	P18089	Alpha-2b adrenergic receptor
16	EGFR	P00533	Epidermal growth factor receptor erbB1
16	KLKB1	P03952	Plasma kallikrein
16	NUDT1	P36639	7,8-dihydro-8-oxoguanine triphosphatase
16	PDE10A	Q9Y233	Phosphodiesterase 10A
16	NISCH	Q9Y2I1	Nischarin
16	CA12	O43570	Carbonic anhydrase XII
16	CA14	Q9ULX7	Carbonic anhydrase XIV
16	CA9	Q16790	Carbonic anhydrase IX
16	GRM5	P41594	Metabotropic glutamate receptor 5 (by homology)
16	TGM2	P21980	Protein-glutamine gamma-glutamyltransferase
17	PTGS1	P23219	Prostaglandin G/H synthase 1
17	PTGS2	P35354	Prostaglandin G/H synthase 2
17	CHRM3	P20309	Muscarinic acetylcholine receptor M3
17	CHRM1	P11229	Muscarinic acetylcholine receptor M1
17	CHRM2	P08172	Muscarinic acetylcholine receptor M2
17	GABRA2	P47869	Gamma-aminobutyric-acid receptor alpha-2 subunit
17	RXRA	P19793	Retinoic acid receptor RXR-alpha
17	ADRA1B	P35368	Alpha-1B adrenergic receptor
17	CHRNA2	Q15822	Neuronal acetylcholine receptor subunit alpha-2
17	GABRA1	P14867	Gamma-aminobutyric acid receptor subunit alpha-1
17	GABRA6	Q16445	Gamma-aminobutyric-acid receptor subunit alpha-6
17	NCOA2	Q15596	Nuclear receptor coactivator 2
17	SLC6A2	P23975	Sodium-dependent noradrenaline transporter
17	SLC6A3	Q01959	Sodium-dependent dopamine transporter
17	ADRA1A	P35348	Alpha-1A adrenergic receptor
17	IL6	P05231	Interleukin-6
17	GLI2	P10070	Zinc finger protein GLI2

17	GLI1	P08151	Zinc finger protein GLI1
17	PPARA	Q07869	Peroxisome proliferator-activated receptor alpha
17	CNR2	P34972	Cannabinoid receptor 2
17	FAAH	O00519	Anandamide amidohydrolase
17	TRPV1	Q8NER1	Transient receptor potential cation channel subfamily V member 1
18	PTGS1	P23219	Prostaglandin G/H synthase 1
18	PTGS2	P35354	Prostaglandin G/H synthase 2
18	CHRM3	P20309	Muscarinic acetylcholine receptor M3
18	CHRM1	P11229	Muscarinic acetylcholine receptor M1
18	CHRM2	P08172	Muscarinic acetylcholine receptor M2
18	GABRA2	P47869	Gamma-aminobutyric-acid receptor alpha-2 subunit
18	SLC6A2	P23975	Sodium-dependent noradrenaline transporter
18	ADRA1A	P35348	Alpha-1A adrenergic receptor
18	ADRA1B	P35368	Alpha-1B adrenergic receptor
18	GABRA1	P14867	Gamma-aminobutyric acid receptor subunit alpha-1
18	NCOA2	Q15596	Nuclear receptor coactivator 2
18	GABRA6	Q16445	Gamma-aminobutyric-acid receptor subunit alpha-6
18	ADRB2	P07550	Beta-2 adrenergic receptor
18	CHRNA2	Q15822	Neuronal acetylcholine receptor subunit alpha-2
18	SLC6A3	Q01959	Sodium-dependent dopamine transporter
18	PPARA	Q07869	Peroxisome proliferator-activated receptor alpha
18	CNR2	P34972	Cannabinoid receptor 2
18	FAAH	O00519	Anandamide amidohydrolase
18	TRPV1	Q8NER1	Transient receptor potential cation channel subfamily V member 1
18	GLI2	P10070	Zinc finger protein GLI2
18	GLI1	P08151	Zinc finger protein GLI1
19	PTGS1	P23219	Prostaglandin G/H synthase 1
19	PTGS2	P35354	Prostaglandin G/H synthase 2
19	SLC6A2	P23975	Sodium-dependent noradrenaline transporter
19	NCOA2	Q15596	Nuclear receptor coactivator 2
19	PPARA	Q07869	Peroxisome proliferator-activated receptor alpha
19	CNR2	P34972	Cannabinoid receptor 2
19	ADORA1	P30542	Adenosine A1 receptor
19	ADORA2A	P29274	Adenosine A2a receptor

19	ADORA3	P0DMS8	Adenosine A3 receptor
19	AR	P10275	Androgen Receptor
19	CYP19A1	P11511	Cytochrome P450 19A1
19	ESR1	P03372	Estrogen receptor alpha
19	ESR2	Q92731	Estrogen receptor beta
19	MAOB	P27338	Monoamine oxidase B
20	PTGS1	P23219	Prostaglandin G/H synthase 1
20	PTGS2	P35354	Prostaglandin G/H synthase 2
20	CHRM3	P20309	Muscarinic acetylcholine receptor M3
20	CHRM1	P11229	Muscarinic acetylcholine receptor M1
20	CHRM2	P08172	Muscarinic acetylcholine receptor M2
20	GABRA2	P47869	Gamma-aminobutyric-acid receptor alpha-2 subunit
20	GABRA1	P14867	Gamma-aminobutyric acid receptor subunit alpha-1
20	RXRA	P19793	Retinoic acid receptor RXR-alpha
20	SLC6A2	P23975	Sodium-dependent noradrenaline transporter
20	ADRA1B	P35368	Alpha-1B adrenergic receptor
20	NCOA2	Q15596	Nuclear receptor coactivator 2
20	GABRA6	Q16445	Gamma-aminobutyric-acid receptor subunit alpha-6
20	CYP19A1	P11511	Cytochrome P450 19A1
20	PPARA	Q07869	Peroxisome proliferator-activated receptor alpha
20	CNR2	P34972	Cannabinoid receptor 2
20	AR	P10275	Androgen Receptor (by homology)
20	ESR1	P03372	Estrogen receptor alpha
20	ACHE	P22303	Acetylcholinesterase
20	SLC6A4	P31645	Serotonin transporter
20	CYP2C19	P33261	Cytochrome P450 2C19
20	NR1H3	Q13133	LXR-alpha
20	BCHE	P06276	Butyrylcholinesterase
20	PTPN1	P18031	Protein-tyrosine phosphatase 1B
20	NR1I3	Q14994	Nuclear receptor subfamily 1 group I member 3 (by homology)
20	FAAH	O00519	Anandamide amidohydrolase
20	TRPV1	Q8NER1	Vanilloid receptor
20	ESR2	Q92731	Estrogen receptor beta
21	PTGS1	P23219	Prostaglandin G/H synthase 1
21	PTGS2	P35354	Prostaglandin G/H synthase 2
21	GABRA2	P47869	Gamma-aminobutyric-acid receptor alpha-2 subunit
21	GABRA6	Q16445	Gamma-aminobutyric-acid receptor subunit

			alpha-6
21	GABRA1	P14867	Gamma-aminobutyric acid receptor subunit alpha-1
21	ADRA1B	P35368	Alpha-1B adrenergic receptor
21	NCOA2	Q15596	Nuclear receptor coactivator 2
21	CHRM2	P08172	Muscarinic acetylcholine receptor M2
21	COQ7	Q99807	5-demethoxyubiquinone hydroxylase, mitochondrial
21	PPARA	Q07869	Peroxisome proliferator-activated receptor alpha
21	CNR2	P34972	Cannabinoid receptor 2
21	FAAH	O00519	Anandamide amidohydrolase
21	TRPV1	Q8NER1	Vanilloid receptor
21	GLI2	P10070	Zinc finger protein GLI2
21	GLI1	P08151	Zinc finger protein GLI1
21	GPR18	Q14330	N-arachidonyl glycine receptor
22	PTGS1	P23219	Prostaglandin G/H synthase 1
22	PTGS2	P35354	Prostaglandin G/H synthase 2
22	CHRM3	P20309	Muscarinic acetylcholine receptor M3
22	CHRM1	P11229	Muscarinic acetylcholine receptor M1
22	GABRA1	P14867	Gamma-aminobutyric acid receptor subunit alpha-1
22	RXRA	P19793	Retinoic acid receptor RXR-alpha
22	SLC6A2	P23975	Sodium-dependent noradrenaline transporter
22	ADRA1B	P35368	Alpha-1B adrenergic receptor
22	NCOA2	Q15596	Nuclear receptor coactivator 2
22	PPARA	Q07869	Peroxisome proliferator-activated receptor alpha
22	CNR2	P34972	Cannabinoid receptor 2
22	NR1H3	Q13133	LXR-alpha
22	CYP19A1	P11511	Cytochrome P450 19A1
23	CHRM1	P11229	Muscarinic acetylcholine receptor M1
23	GABRA1	P14867	Gamma-aminobutyric acid receptor subunit alpha-1
23	CYP17A1	P05093	Cytochrome P450 17A1
23	CYP19A1	P11511	Cytochrome P450 19A1
23	RORC	P51449	Nuclear receptor ROR-gamma
23	SREBF2	Q12772	Sterol regulatory element-binding protein 2
23	NPC1L1	Q9UHC9	Niemann-Pick C1-like protein 1
23	CYP51A1	Q16850	Cytochrome P450 51 (by homology)
23	NR1H3	Q13133	LXR-alpha
23	AR	P10275	Androgen Receptor
23	ESR1	P03372	Estrogen receptor alpha

23	HMGCR	P04035	HMG-CoA reductase
23	SHBG	P04278	Testis-specific androgen-binding protein
23	ESR2	Q92731	Estrogen receptor beta
23	RORA	P35398	Nuclear receptor ROR-alpha
23	NR1I3	Q14994	Nuclear receptor subfamily 1 group I member 3 (by homology)
23	G6PD	P11413	Glucose-6-phosphate 1-dehydrogenase
23	CHRM2	P08172	Muscarinic acetylcholine receptor M2
23	ACHE	P22303	Acetylcholinesterase
23	SLC6A2	P23975	Norepinephrine transporter
23	SLC6A4	P31645	Serotonin transporter
23	CYP2C19	P33261	Cytochrome P450 2C19
23	BCHE	P06276	Butyrylcholinesterase
23	UGT2B7	P16662	UDP-glucuronosyltransferase 2B7
23	IDO1	P14902	Indoleamine 2,3-dioxygenase
23	SQLE	Q14534	Squalene monooxygenase
23	SERPINA6	P08185	Corticosteroid binding globulin
23	NR1H2	P55055	LXR-beta
23	PTGER2	P43116	Prostanoid EP2 receptor (by homology)
23	PPARA	Q07869	Peroxisome proliferator-activated receptor alpha
23	PPARD	Q03181	Peroxisome proliferator-activated receptor delta
23	HSD11B1	P28845	11-beta-hydroxysteroid dehydrogenase 1
23	VDR	P11473	Vitamin D receptor
23	PTPN1	P18031	Protein-tyrosine phosphatase 1B
23	CES2	O00748	Carboxylesterase 2
23	TBXAS1	P24557	Thromboxane-A synthase
23	PTPN6	P29350	Protein-tyrosine phosphatase 1C
23	PTPN2	P17706	T-cell protein-tyrosine phosphatase
23	PTGES	O14684	Prostaglandin E synthase
24	PPARA	Q07869	Peroxisome proliferator-activated receptor alpha
24	CNR2	P34972	Cannabinoid receptor 2
24	TRPV1	Q8NER1	Vanilloid receptor
24	GLI2	P10070	Zinc finger protein GLI2
24	GLI1	P08151	Zinc finger protein GLI1
25	PTGS2	P35354	Prostaglandin G/H synthase 2
25	RXRA	P19793	Retinoic acid receptor RXR-alpha
25	GABRA1	P14867	Gamma-aminobutyric acid receptor subunit alpha-1
25	SLC6A2	P23975	Sodium-dependent noradrenaline transporter
25	MAOB	P27338	Amine oxidase [flavin-containing] B

25	GLI2	P10070	Zinc finger protein GLI2
25	GLI1	P08151	Zinc finger protein GLI1
25	PPARA	Q07869	Peroxisome proliferator-activated receptor alpha
25	CNR2	P34972	Cannabinoid receptor 2
25	TRPV1	Q8NER1	Vanilloid receptor
25	ADORA1	P30542	Adenosine A1 receptor
25	ADORA2A	P29274	Adenosine A2a receptor
25	ADORA3	P0DMS8	Adenosine A3 receptor
25	PTPN2	P17706	T-cell protein-tyrosine phosphatase
26	PTGS2	P35354	Prostaglandin G/H synthase 2
26	SLC6A2	P23975	Sodium-dependent noradrenaline transporter
26	GABRA1	P14867	Gamma-aminobutyric acid receptor subunit alpha-1
26	IDO1	P14902	Indoleamine 2,3-dioxygenase
26	PER2	O15055	Period circadian protein homolog 2
26	BACE1	P56817	Beta-secretase 1
26	GCGR	P47871	Glucagon receptor
26	PGR	P06401	Progesterone receptor
26	GRM5	P41594	Metabotropic glutamate receptor 5
26	PSEN2	P49810	Presenilin-2
26	NCSTN	Q92542	Nicastrin
26	PSENEN	Q9NZ42	Gamma-secretase subunit PEN-2
26	APH1A	Q96BI3	Gamma-secretase subunit APH-1A
26	PSEN1	P49768	P49768
26	APH1B	Q8WW43	Gamma-secretase subunit APH-1B
26	HSD17B2	P37059	Estradiol 17-beta-dehydrogenase 2
26	NR3C1	P04150	Glucocorticoid receptor
26	SLC6A3	Q01959	Dopamine transporter (by homology)
26	KCNH2	Q12809	HERG
26	SLC10A2	Q12908	Ileal bile acid transporter
26	AVPR1A	P37288	Vasopressin V1a receptor
26	NPY5R	Q15761	Neuropeptide Y receptor type 5
26	TTL	Q8NG68	Tubulin--tyrosine ligase
26	NR1H3	Q13133	LXR-alpha
26	CYP2C9	P11712	Cytochrome P450 2C9
26	CYP3A4	P08684	Cytochrome P450 3A4
26	MAPK8	P45983	c-Jun N-terminal kinase 1
26	PGGT1B	P53609	Geranylgeranyl transferase type I beta subunit
26	PABPC1	P11940	Polyadenylate-binding protein 1
26	MAPK14	Q16539	MAP kinase p38 alpha (by homology)
26	GRM2	Q14416	Metabotropic glutamate receptor 2
26	PRKCD	Q05655	Protein kinase C delta

26	TRPV1	Q8NER1	Vanilloid receptor
26	AKR1C3	P42330	Aldo-keto-reductase family 1 member C3
26	CDC25A	P30304	Dual specificity phosphatase Cdc25A
26	F2R	P25116	Proteinase-activated receptor 1
26	CCR1	P32246	C-C chemokine receptor type 1
26	PDE2A	O00408	Phosphodiesterase 2A
26	PDE10A	Q9Y233	Phosphodiesterase 10A
26	IL6ST	P40189	Interleukin-6 receptor subunit beta
26	CYP11B1	P15538	Cytochrome P450 11B1
26	CYP11B2	P19099	Cytochrome P450 11B2
26	NR1H2	P55055	LXR-beta
26	SCN9A	Q15858	Sodium channel protein type IX alpha subunit
26	JAK1	P23458	Tyrosine-protein kinase JAK1
26	JAK2	O60674	Tyrosine-protein kinase JAK2
26	JAK3	P52333	Tyrosine-protein kinase JAK3
26	PYGL	P06737	Liver glycogen phosphorylase
26	KCNA5	P22460	Voltage-gated potassium channel subunit Kv1.5
26	KCNA3	P22001	Voltage-gated potassium channel subunit Kv1.3
26	CASR	P41180	Calcium sensing receptor
26	EPHX2	P34913	Epoxide hydratase
26	CHRM4	P08173	Muscarinic acetylcholine receptor M4
26	CHRM5	P08912	Muscarinic acetylcholine receptor M5
26	BRS3	P32247	Bombesin receptor subtype-3
26	PRCP	P42785	Lysosomal Pro-X carboxypeptidase
26	NPY2R	P49146	Neuropeptide Y receptor type 2
26	TYK2	P29597	Tyrosine-protein kinase TYK2
26	LYPLA2	O95372	Acyl-protein thioesterase 2
26	OXTR	P30559	Oxytocin receptor (by homology)
26	PRKCG	P05129	Protein kinase C gamma
26	PRKCA	P17252	Protein kinase C alpha
26	PRKCB	P05771	Protein kinase C beta
26	PRKCE	Q02156	Protein kinase C epsilon
26	PRKCH	P24723	Protein kinase C eta
26	PRKCQ	Q04759	Protein kinase C theta
26	HSD17B3	P37058	Estradiol 17-beta-dehydrogenase 3
26	RASGRP1	O95267	RAS guanyl-releasing protein 1 (by homology)
26	PTPN1	P18031	Protein-tyrosine phosphatase 1B
26	NOS1	P29475	Nitric-oxide synthase, brain
26	LYPLA1	O75608	Acyl-protein thioesterase 1
26	SLC6A9	P48067	Glycine transporter 1 (by homology)

26	FDFT1	P37268	Squalene synthetase
26	GCK	P35557	Hexokinase type IV
26	GABBR2	O75899	Gamma-aminobutyric acid type B receptor subunit 2
26	GABBR1	Q9UBS5	Gamma-aminobutyric acid type B receptor subunit 1
26	ABHD6	Q9BV23	Monoacylglycerol lipase ABHD6
26	ICMT	O60725	Isoprenylcysteine carboxyl methyltransferase
26	CSF1R	P07333	Macrophage colony stimulating factor receptor
26	KIT	P10721	Stem cell growth factor receptor
26	C5AR1	P21730	C5a anaphylatoxin chemotactic receptor
26	MDM2	Q00987	p53-binding protein Mdm-2
26	KDR	P35968	Vascular endothelial growth factor receptor 2
26	PGGT1B	P53609	Geranylgeranyl transferase type-1 subunit beta
26	CHRM1	P11229	Muscarinic acetylcholine receptor M1
26	CHRM3	P20309	Muscarinic acetylcholine receptor M3
26	TRPA1	O75762	Transient receptor potential cation channel subfamily A member 1
26	HRH3	Q9Y5N1	Histamine H3 receptor
26	SIGMAR1	Q99720	Sigma opioid receptor
26	EIF2AK1	Q9BQI3	Eukaryotic translation initiation factor 2-alpha kinase 1
26	GGPS1	O95749	Geranylgeranyl pyrophosphate synthase
26	LSS	P48449	Lanosterol synthase
26	FNTB	P49356	Protein farnesyltransferase subunit beta
26	FNTA	P49354	Protein farnesyltransferase/geranylgeranyltransferase type-1 subunit alpha
27	CHRM1	P11229	Muscarinic acetylcholine receptor M1
27	CHRM3	P20309	Muscarinic acetylcholine receptor M3
27	CHRM2	P08172	Muscarinic acetylcholine receptor M2
27	GABRA1	P14867	Gamma-aminobutyric acid receptor subunit alpha-1
27	UGT2B7	P16662	UDP-glucuronosyltransferase 2B7
27	HSD11B1	P28845	11-beta-hydroxysteroid dehydrogenase 1
27	IDO1	P14902	Indoleamine 2,3-dioxygenase
27	PTGS1	P23219	Cyclooxygenase-1
27	NR1H3	Q13133	LXR-alpha
27	SLC6A3	Q01959	Dopamine transporter (by homology)
27	PGR	P06401	Progesterone receptor
28	CHRM1	P11229	Muscarinic acetylcholine receptor M1
28	CHRM3	P20309	Muscarinic acetylcholine receptor M3

28	CHRM2	P08172	Muscarinic acetylcholine receptor M2
28	ACHE	P22303	Acetylcholinesterase
28	ADRA1B	P35368	Alpha-1B adrenergic receptor
28	DPP4	P27487	Dipeptidyl peptidase IV
28	NCOA2	Q15596	Nuclear receptor coactivator 2
29	CHRM3	P20309	Muscarinic acetylcholine receptor M3
29	CHRM2	P08172	Muscarinic acetylcholine receptor M2
29	UGT2B7	P16662	UDP-glucuronosyltransferase 2B7
29	HSD11B1	P28845	11-beta-hydroxysteroid dehydrogenase 1
29	IDO1	P14902	Indoleamine 2,3-dioxygenase
29	PTGS1	P23219	Cyclooxygenase-1
29	NR1H3	Q13133	LXR-alpha
29	SLC6A3	Q01959	Dopamine transporter (by homology)
29	PGR	P06401	Progesterone receptor
30	CHRM1	P11229	Muscarinic acetylcholine receptor M1
30	CHRM3	P20309	Muscarinic acetylcholine receptor M3
30	GABRA2	P47869	Gamma-aminobutyric-acid receptor alpha-2 subunit
30	GABRA1	P14867	Gamma-aminobutyric acid receptor subunit alpha-1
30	SLC6A2	P23975	Sodium-dependent noradrenaline transporter
30	ADRA1B	P35368	Alpha-1B adrenergic receptor
30	GABRA6	Q16445	Gamma-aminobutyric-acid receptor subunit alpha-6
30	CHRNA7	P36544	Neuronal acetylcholine receptor protein, alpha-7 chain
30	CYP19A1	P11511	Cytochrome P450 19A1
30	BCHE	P06276	Butyrylcholinesterase
30	PTPN1	P18031	Protein-tyrosine phosphatase 1B
30	NR1H3	Q13133	LXR-alpha
30	CYP17A1	P05093	Cytochrome P450 17A1
30	CHRM2	P08172	Muscarinic acetylcholine receptor M2
30	AR	P10275	Androgen Receptor (by homology)
30	ESR1	P03372	Estrogen receptor alpha
30	ACHE	P22303	Acetylcholinesterase
30	SLC6A4	P31645	Norepinephrine transporter
30	CYP2C19	P33261	Serotonin transporter
30	RORC	P51449	Cytochrome P450 2C19
30	SREBF2	Q12772	Nuclear receptor ROR-gamma
30	NPC1L1	Q9UHC9	Sterol regulatory element-binding protein 2
30	CYP51A1	Q16850	Niemann-Pick C1-like protein 1
30	HMGCR	P04035	Cytochrome P450 51 (by homology)
30	NR1I3	Q14994	HMG-CoA reductase

30	ESR2	Q92731	Nuclear receptor subfamily 1 group I member 3 (by homology)
30	SHBG	P04278	Estrogen receptor beta
30	HSD11B1	P28845	Testis-specific androgen-binding protein
30	SQLE	Q14534	11-beta-hydroxysteroid dehydrogenase 1
30	POLB	P06746	Squalene monooxygenase
30	PDE4D	Q08499	DNA polymerase beta
30	CES2	O00748	Phosphodiesterase 4D
30	PTPRF	P10586	Carboxylesterase 2
30	PTPN2	P17706	Receptor-type tyrosine-protein phosphatase F (LAR)
30	PLA2G1B	P04054	T-cell protein-tyrosine phosphatase
30	ACP1	P24666	Phospholipase A2 group 1B
30	AKR1B10	O60218	Low molecular weight phosphotyrosine protein phosphatase
30	UGT2B7	P16662	Aldo-keto reductase family 1 member B10
30	ATP12A	P54707	UDP-glucuronosyltransferase 2B7
30	PTPN6	P29350	Potassium-transporting ATPase alpha chain 2
30	CDC25B	P30305	Protein-tyrosine phosphatase 1C
30	RORA	P35398	Dual specificity phosphatase Cdc25B
30	CD81	P60033	Nuclear receptor ROR-alpha
30	G6PD	P11413	CD81 antigen
30	PREP	P48147	Glucose-6-phosphate 1-dehydrogenase
30	FABP4	P15090	Prolyl endopeptidase
30	PPARG	P37231	Fatty acid binding protein adipocyte
30	PPARA	Q07869	Peroxisome proliferator-activated receptor gamma
30	TERT	O14746	Peroxisome proliferator-activated receptor alpha
30	FABP3	P05413	Telomerase reverse transcriptase
30	FABP5	Q01469	Fatty acid binding protein muscle
30	HSD11B2	P80365	Fatty acid binding protein epidermal
30	PPARD	Q03181	11-beta-hydroxysteroid dehydrogenase 2
30	FABP1	P07148	Peroxisome proliferator-activated receptor delta
30	FNTA	P49354	Protein farnesyltransferase/geranylgeranyltransferase type-1 subunit alpha
30	PTPN11	Q06124	Protein farnesyltransferase
30	FNTB	P49356	Protein farnesyltransferase subunit beta
31	CHRM1	P11229	Muscarinic acetylcholine receptor M1
31	GABRA2	P47869	Gamma-aminobutyric-acid receptor alpha-2 subunit

31	GABRA1	P14867	Gamma-aminobutyric acid receptor subunit alpha-1
31	SLC6A2	P23975	Sodium-dependent noradrenaline transporter
31	GABRA6	Q16445	Gamma-aminobutyric-acid receptor subunit alpha-6
31	CHRNA7	P36544	Neuronal acetylcholine receptor protein, alpha-7 chain
31	CYP19A1	P11511	Cytochrome P450 19A1
31	BCHE	P06276	Butyrylcholinesterase
31	PTPN1	P18031	Protein-tyrosine phosphatase 1B
31	NR1H3	Q13133	LXR-alpha
31	CYP17A1	P05093	Cytochrome P450 17A1
31	CHRM2	P08172	Muscarinic acetylcholine receptor M2
31	AR	P10275	Androgen Receptor (by homology)
31	ESR1	P03372	Estrogen receptor alpha
31	ACHE	P22303	Acetylcholinesterase
31	SLC6A4	P31645	Serotonin transporter
31	CYP2C19	P33261	Cytochrome P450 2C19
31	RORC	P51449	Nuclear receptor ROR-gamma
31	SREBF2	Q12772	Sterol regulatory element-binding protein 2
31	NPC1L1	Q9UHC9	Niemann-Pick C1-like protein 1
31	CYP51A1	Q16850	Cytochrome P450 51 (by homology)
31	HMGCR	P04035	HMG-CoA reductase
31	NR1I3	Q14994	Nuclear receptor subfamily 1 group I member 3 (by homology)
31	ESR2	Q92731	Estrogen receptor beta
31	SHBG	P04278	Testis-specific androgen-binding protein
31	HSD11B1	P28845	11-beta-hydroxysteroid dehydrogenase 1
31	SQLE	Q14534	Squalene monooxygenase
31	POLB	P06746	DNA polymerase beta
31	PDE4D	Q08499	Phosphodiesterase 4D
31	CES2	O00748	Carboxylesterase 2
31	PTPRF	P10586	Receptor-type tyrosine-protein phosphatase F (LAR)
31	PTPN2	P17706	T-cell protein-tyrosine phosphatase
31	PLA2G1B	P04054	Phospholipase A2 group 1B
31	ACP1	P24666	Low molecular weight phosphotyrosine protein phosphatase
31	AKR1B10	O60218	Aldo-keto reductase family 1 member B10
31	UGT2B7	P16662	UDP-glucuronosyltransferase 2B7
31	ATP12A	P54707	Potassium-transporting ATPase alpha chain 2
31	PTPN6	P29350	Protein-tyrosine phosphatase 1C
31	CDC25B	P30305	Dual specificity phosphatase Cdc25B

31	RORA	P35398	Nuclear receptor ROR-alpha
31	CD81	P60033	CD81 antigen
31	G6PD	P11413	Glucose-6-phosphate 1-dehydrogenase
31	PREP	P48147	Prolyl endopeptidase
31	FABP4	P15090	Fatty acid binding protein adipocyte
31	PPARG	P37231	Peroxisome proliferator-activated receptor gamma
31	PPARA	Q07869	Peroxisome proliferator-activated receptor alpha
31	TERT	O14746	Telomerase reverse transcriptase
31	FABP3	P05413	Fatty acid binding protein muscle
31	FABP5	Q01469	Fatty acid binding protein epidermal
31	HSD11B2	P80365	11-beta-hydroxysteroid dehydrogenase 2
31	PPARD	Q03181	Peroxisome proliferator-activated receptor delta
31	FABP1	P07148	Fatty acid-binding protein, liver
31	FNTA	P49354	Protein farnesyltransferase/geranylgeranyltransferase type-1 subunit alpha
31	FNTB	P49356	Protein farnesyltransferase subunit beta
31	PTPN11	Q06124	Protein-tyrosine phosphatase 2C
33	SLC6A2	P23975	Sodium-dependent noradrenaline transporter
33	MAOB	P27338	Amine oxidase [flavin-containing] B
33	PTGS2	P35354	Prostaglandin G/H synthase 2
33	SQLE	Q14534	Squalene monooxygenase
33	GGPS1	O95749	Geranylgeranyl pyrophosphate synthase
33	LSS	P48449	Lanosterol synthase
33	BTN3A1	FNTB	Butyrophilin subfamily 3 member A1
33	FNTB	P49356	Protein farnesyltransferase subunit beta
33	FNTA	P49354	Protein farnesyltransferase/geranylgeranyltransferase type-1 subunit alpha
33	CDC25C	P30307	M-phase inducer phosphatase 3
33	FDFT1	P37268	Squalene synthase
34	PTGS2	P35354	Prostaglandin G/H synthase 2
34	SLC6A2	P23975	Sodium-dependent noradrenaline transporter
34	MAOB	P27338	Amine oxidase [flavin-containing] B
34	NCOA2	Q15596	Nuclear receptor coactivator 2
34	PTGS1	P23219	Prostaglandin G/H synthase 1
34	RXRA	P19793	Retinoic acid receptor RXR-alpha
34	IL6R	P08887	Interleukin-6
34	CASP3	P42574	Caspase-3
34	IVL	P07476	Involucrin

34	RASGRF2	O14827	Ras-specific guanine nucleotide-releasing factor 2
34	BAK1	Q16611	Bcl-2 homologous antagonist/killer
34	TLR4	O00206	Toll-like receptor 4
34	LPL	P06858	Lipoprotein lipase
34	HMGCR	P04035	3-hydroxy-3-methylglutaryl-coenzyme A reductase
34	PPARA	Q07869	Peroxisome proliferator-activated receptor alpha
34	TIR2	O60603	Toll-like receptor 2
34	DEFB4A	O15263	Beta-defensin 2
34	HIRA	P54198	Protein HIRA
34	SERTAD3	Q9UJW9	SERTA domain-containing protein 3
34	PGR	P06401	Progesterone receptor
34	NR1H4	Q96RI1	Bile acid receptor
34	GLS2	Q9UI32	Glutaminase liver isoform, mitochondrial
34	IGHD	P01880	Ig delta chain C region
34	AKR1B10	O60218	Aldo-keto reductase family 1 member B10
34	UGT2B4	P06133	UDP-glucuronosyltransferase 2B4
34	AKR1C3	P42330	Aldo-keto reductase family 1 member C3
34	UGT1A1	P22309	UDP-glucuronosyltransferase 1A1
34	UGT1A3	P35503	UDP-glucuronosyltransferase 1A3
34	UGT1A4	P22310	UDP-glucuronosyltransferase 1A4
34	UGT1A9	O60656	UDP-glucuronosyltransferase 1A9
34	SQLE	Q14534	Squalene monooxygenase
34	GGPS1	O95749	Geranylgeranyl pyrophosphate synthase
34	LSS	P48449	Lanosterol synthase
34	BTN3A1	FNTB	Butyrophilin subfamily 3 member A1
34	FNTB	P49356	Protein farnesyltransferase subunit beta
34	FNTA	P49354	Protein farnesyltransferase/geranylgeranyltransferase type-1 subunit alpha
34	CDC25C	P30307	M-phase inducer phosphatase 3
34	FDFT1	P37268	Squalene synthase
34	CNR1	P21554	Cannabinoid receptor 1 (by homology)
34	CNR2	P34972	Cannabinoid receptor 2
34	GCGR	P47871	Glucagon receptor
34	PSEN2	P49810	Presenilin-2
34	PSENEN	Q9NZ42	Gamma-secretase subunit PEN-2
34	NCSTN	Q92542	Nicastrin
34	APH1A	Q96BI3	Gamma-secretase subunit APH-1A
34	PSEN1	P49768	Presenilin-1
34	APH1B	Q8WW43	Gamma-secretase subunit APH-1B

34	PER2	O15055	Period circadian protein homolog 2
34	PRKCD	Q05655	Protein kinase C delta
34	PRKCA	P17252	Protein kinase C alpha
34	HSD11B1	P28845	11-beta-hydroxysteroid dehydrogenase 1
34	UGT2B7	P16662	UDP-glucuronosyltransferase 2B7
34	BACE1	P56817	Beta-secretase 1
34	G6PD	P11413	Glucose-6-phosphate 1-dehydrogenase
34	TRPA1	O75762	Transient receptor potential cation channel subfamily A member 1
34	HSD11B2	P80365	11-beta-hydroxysteroid dehydrogenase 2
34	GABBR1	Q9UBS5	GABA-B receptor (by homology)
34	TBXAS1	P24557	Thromboxane-A synthase
34	EPHX2	P34913	Epoxide hydratase
34	CHRM4	P08173	Muscarinic acetylcholine receptor M4
34	CHRM5	P08912	Muscarinic acetylcholine receptor M5
34	GRM5	P41594	Metabotropic glutamate receptor 5
34	PABPC1	P11940	Polyadenylate-binding protein 1
34	AR	P10275	Androgen Receptor
34	JAK2	O60674	Tyrosine-protein kinase JAK2
34	KCNA3	P22001	Voltage-gated potassium channel subunit Kv1.3
34	IDO1	P14902	Indoleamine 2,3-dioxygenase
34	KCNH2	Q12809	HERG
34	CYP11B2	P19099	Cytochrome P450 11B2
34	NR1H2	P55055	LXR-beta
34	MDM2	Q00987	p53-binding protein Mdm-2
34	SHBG	P04278	Testis-specific androgen-binding protein
34	C5AR1	P21730	C5a anaphylatoxin chemotactic receptor
34	PRKCG	P05129	Protein kinase C gamma
34	PRKCB	P05771	Protein kinase C beta
34	PRKCE	Q02156	Protein kinase C epsilon
34	PRKCH	P24723	Protein kinase C eta
34	PRKCQ	Q04759	Protein kinase C theta
34	RASGRP1	O95267	RAS guanyl-releasing protein 1 (by homology)
34	GPR88	Q9GZN0	Probable G-protein coupled receptor 88
34	CYP11B1	P15538	Cytochrome P450 11B1
34	CHRM1	P11229	Muscarinic acetylcholine receptor M1
34	CHRM3	P20309	Muscarinic acetylcholine receptor M3
34	SIGMAR1	Q99720	Sigma opioid receptor
34	BRS3	P32247	Bombesin receptor subtype-3
34	JAK1	P23458	Tyrosine-protein kinase JAK1
34	PLA2G7	Q13093	LDL-associated phospholipase A2

34	STS	P08842	Steryl-sulfatase
34	LIPE	Q05469	Hormone sensitive lipase
35	PTGS1	P23219	Prostaglandin G/H synthase 1
35	NCOA2	Q15596	Nuclear receptor coactivator 2
35	IGHG1	P01857	Ig gamma-1 chain C region
35	RELA	Q04206	Transcription factor p65
35	IL10	P22301	Interleukin-10
35	TNF	P01375	Tumor necrosis factor
35	IL6	P05231	Interleukin-6
35	PTGS2	P35354	Prostaglandin G/H synthase 2
35	PTGER3	P43115	Prostaglandin E2 receptor EP3 subtype
35	FFAR1	O14842	Free fatty acid receptor 1
35	AKR1B10	O60218	Aldo-keto reductase family 1 member B10
35	CA1	P00915	Carbonic anhydrase I
35	CA2	P00918	Carbonic anhydrase II
35	HMGCR	P04035	HMG-CoA reductase
35	SHBG	P04278	Testis-specific androgen-binding protein
35	FABP3	P05413	Fatty acid binding protein muscle
35	POLB	P06746	DNA polymerase beta (by homology)
35	SERPINA6	P08185	Corticosteroid binding globulin
35	ALOX5	P09917	Arachidonate 5-lipoxygenase
35	G6PD	P11413	Glucose-6-phosphate 1-dehydrogenase
35	VDR	P11473	Vitamin D receptor
35	CYP19A1	P11511	Cytochrome P450 19A1
35	FABP2	P12104	Fatty acid binding protein intestinal
35	FABP4	P15090	Fatty acid binding protein adipocyte
35	UGT2B7	P16662	UDP-glucuronosyltransferase 2B7
35	PTPN1	P18031	Protein-tyrosine phosphatase 1B
35	F2R	P25116	Proteinase-activated receptor 1
35	DCK	P27707	Deoxycytidine kinase
35	HSD11B1	P28845	11-beta-hydroxysteroid dehydrogenase 1
35	HSD17B3	P37058	Estradiol 17-beta-dehydrogenase 3
35	KDM5C	P41229	Lysine-specific demethylase 5C
35	SLC1A1	P43005	Excitatory amino acid transporter 3
35	PTGFR	P43088	Prostanoid FP receptor
35	CPT1A	P50416	Carnitine O-palmitoyltransferase 1, liver isoform (by homology)
35	FABP5	Q01469	Fatty acid binding protein epidermal
35	PPARD	Q03181	Peroxisome proliferator-activated receptor delta
35	PPARA	Q07869	Peroxisome proliferator-activated receptor alpha
35	PFKFB3	Q16875	6-phosphofructo-2-kinase/fructose-2,6-

			bisphosphatase 3
35	MPEG1	Q2M385	Macrophage-expressed gene 1 protein
35	SLC22A6	Q4U2R8	Solute carrier family 22 member 6 (by homology)
35	TRPV1	Q8NER1	Vanilloid receptor
35	GPBAR1	Q8TDU6	G-protein coupled bile acid receptor 1
35	NR1H4	Q96RI1	Bile acid receptor FXR
35	GABBR1	Q9UBS5	GABA-B receptor (by homology)
35	PHF8	Q9UPP1	Histone lysine demethylase PHF8
35	KDM2A	Q9Y2K7	Lysine-specific demethylase 2A
35	ACER2	Q5QJU3	Alkaline ceramidase 2
35	ACP1	P24666	Low molecular weight phosphotyrosine protein phosphatase
35	ADH1A	P07327	Alcohol dehydrogenase 1A
35	ADH1B	P00325	All-trans-retinol dehydrogenase [NAD(+)] ADH1B
35	ADH1C	P00326	Alcohol dehydrogenase 1C
35	ADH7	P40394	All-trans-retinol dehydrogenase [NAD(+)] ADH7
35	APEX1	P27695	DNA-(apurinic or apyrimidinic site) lyase
35	ASAH1	Q13510	Acid ceramidase
35	CA14	Q9ULX7	Carbonic anhydrase 14
35	CDC25A	P30304	M-phase inducer phosphatase 1
35	CDC25B	P30305	M-phase inducer phosphatase 2
35	CDC25C	P30307	M-phase inducer phosphatase 3
35	CERT1	Q9Y5P4	Ceramide transfer protein
35	CES1	P23141	Liver carboxylesterase 1
35	CES2	O00748	Cocaine esterase
35	CNR1	P21554	Cannabinoid receptor 1
35	CNR2	P34972	Cannabinoid receptor 2
35	DAGLA	Q9Y4D2	Sn1-specific diacylglycerol lipase alpha
35	DNM1	Q05193	Dynamin-1
35	ENPP2	Q13822	Ectonucleotide pyrophosphatase/phosphodiesterase family member 2
35	EPHX1	P07099	Epoxide hydrolase 1
35	EPHX2	P34913	Bifunctional epoxide hydrolase 2
35	FAAH	O00519	Fatty-acid amide hydrolase 1
35	FDPS	P14324	Farnesyl pyrophosphate synthase
35	FUT7	Q11130	Alpha-(1,3)-fucosyltransferase 7
35	GBA	P04062	Lysosomal acid glucosylceramidase
35	GBA2	Q9HCG7	Non-lysosomal glucosylceramidase
35	GGPS1	O95749	Geranylgeranyl pyrophosphate synthase

35	GNAI1	P63096	Guanine nucleotide-binding protein G(i) subunit alpha-1
35	GNAI3	P08754	Guanine nucleotide-binding protein G(i) subunit alpha-3
35	GNAO1	P09471	Guanine nucleotide-binding protein G(o) subunit alpha
35	GPR174	Q9BXC1	Probable G-protein coupled receptor 174
35	GPR18	Q14330	N-arachidonyl glycine receptor
35	GPR34	Q9UPC5	Probable G-protein coupled receptor 34
35	IARS1	P41252	Isoleucine--tRNA ligase, cytoplasmic
35	KAT2B	Q92831	Histone acetyltransferase KAT2B
35	KDM5A	P29375	Lysine-specific demethylase 5A
35	LPAR1	Q92633	Lysophosphatidic acid receptor 1
35	LPAR2	Q9HBW0	Lysophosphatidic acid receptor 2
35	LPAR3	Q9UBY5	Lysophosphatidic acid receptor 3
35	LPAR4	Q99677	Lysophosphatidic acid receptor 4
35	LPAR5	Q9H1C0	Lysophosphatidic acid receptor 5
35	LPAR6	P43657	Lysophosphatidic acid receptor 6
35	LY96	Q9Y6Y9	Lymphocyte antigen 96
35	NAAA	Q02083	N-acylethanolamine-hydrolyzing acid amidase
35	NOD1	Q9Y239	Nucleotide-binding oligomerization domain-containing protein 1
35	OXER1	Q8TDS5	Oxoeicosanoid receptor 1
35	P2RY10	O00398	Putative P2Y purinoceptor 10
35	PAM	P19021	Peptidyl-glycine alpha-amidating monooxygenase
35	PAOX	Q6QHF9	Peroxisomal N(1)-acetyl-spermine/spermidine oxidase
35	PGA5	P0DJD9	Pepsin A-5
35	PLA2G10	O15496	Group 10 secretory phospholipase A2
35	PLA2G2A	P14555	Phospholipase A2, membrane associated
35	PLA2G2C	Q5R387	Putative inactive group IIC secretory phospholipase A2
35	PLA2G4B	P0C869	Cytosolic phospholipase A2 beta
35	PLA2G5	P39877	Phospholipase A2 group V
35	PLCG2	P16885	1-phosphatidylinositol 4,5-bisphosphate phosphodiesterase gamma-2
35	POLA1	P09884	DNA polymerase alpha catalytic subunit
35	POLH	Q9Y253	DNA polymerase eta
35	POLK	Q9UBT6	DNA polymerase kappa
35	POLL	Q9UGP5	DNA polymerase lambda
35	POLM	Q9NP87	DNA-directed DNA/RNA polymerase mu

35	PPARG	P37231	Peroxisome proliferator-activated receptor gamma
35	PRKCA	P17252	Protein kinase C alpha type
35	PTPN13	Q12923	Tyrosine-protein phosphatase non-receptor type 13
35	PTPRC	P08575	Receptor-type tyrosine-protein phosphatase C
35	RARB	P10826	Retinoic acid receptor beta
35	S1PR2	O95136	Sphingosine 1-phosphate receptor 2
35	S1PR3	Q99500	Sphingosine 1-phosphate receptor 3
35	S1PR4	O95977	Sphingosine 1-phosphate receptor 4
35	SELP	P16109	P-selectin
35	SLC22A2	O15244	Solute carrier family 22 member 2
35	SLC22A8	Q8TCC7	Solute carrier family 22 member 8
35	SLC25A20	O43772	Mitochondrial carnitine/acylcarnitine carrier protein
35	SMPD2	O60906	Sphingomyelin phosphodiesterase 2
35	SPHK1	Q9NYA1	Sphingosine kinase 1
35	THRA	P10827	Thyroid hormone receptor alpha
35	THRB	P10828	Thyroid hormone receptor beta
35	TLR2	O60603	Toll-like receptor 2
35	TLR4	O00206	Toll-like receptor 4
35	TOP2A	P11388	DNA topoisomerase 2-alpha
35	VEGFA	P15692	Vascular endothelial growth factor A
37	CHRM1	P11229	Muscarinic acetylcholine receptor M1
37	CHRM3	P20309	Muscarinic acetylcholine receptor M3
37	SLC6A2	P23975	Sodium-dependent noradrenaline transporter
37	PTGS1	P23219	Prostaglandin G/H synthase 1
37	PTGS2	P35354	Prostaglandin G/H synthase 2
37	SLC6A3	Q01959	Sodium-dependent dopamine transporter
37	ADRB2	P07550	Beta-2 adrenergic receptor
37	SLC6A4	P31645	Sodium-dependent serotonin transporter
37	NOS3	P29474	Nitric-oxide synthase, endothelial
37	RXRA	P19793	Retinoic acid receptor RXR-alpha
37	GSK3B	P49841	Glycogen synthase kinase-3 beta
37	HSP90AB1	P08238	Heat shock protein HSP 90-beta
37	DPEP1	P16444	Beta-lactamase
37	PDE3A	Q14432	CGMP-inhibited 3',5'-cyclic phosphodiesterase A
37	ADRA1A	P35348	Alpha-1A adrenergic receptor
37	CHRM2	P08172	Muscarinic acetylcholine receptor M2
37	IGHG1	P01857	Ig gamma-1 chain C region
37	HTR2A	P28223	5-hydroxytryptamine 2A receptor
37	GABRA1	P14867	Gamma-aminobutyric acid receptor subunit

			alpha-1
37	GABRA6	Q16445	Gamma-aminobutyric-acid receptor subunit alpha-6
37	CHRNA7	P36544	Neuronal acetylcholine receptor subunit alpha-7
37	TPO	P07202	Thyroid peroxidase
37	PLA2G1B	P04054	Phospholipase A2
37	NR1I3	Q14994	Nuclear receptor subfamily 1 group I member 3
37	HDAC6	Q9UBN7	Histone deacetylase 6
37	NR1I2	O75469	Nuclear receptor subfamily 1 group I member 2
37	SRC	P12931	Proto-oncogene tyrosine-protein kinase Src
37	VEGFA	P15692	Vascular endothelial growth factor A
37	AR	P10275	Androgen receptor
37	ESR1	P03372	Estrogen receptor
37	MEN1	O00255	Menin
37	PDE2A	O00408	cGMP-dependent 3',5'-cyclic phosphodiesterase
37	FAAH	O00519	Fatty-acid amide hydrolase 1
37	HCRTR1	O43613	Orexin receptor type 1
37	HCRTR2	O43614	Orexin receptor type 2
37	GALR3	O60755	Galanin receptor type 3
37	CCNE2	O96020	G1/S-specific cyclin-E2
37	CDK2	P24941	Cyclin-dependent kinase 2
37	CCNE1	P24864	G1/S-specific cyclin-E1
37	ALDH1A1	P00352	Retinal dehydrogenase 1
37	F2	P00734	Prothrombin
37	PLG	P00747	Plasminogen
37	PLAU	P00749	Urokinase-type plasminogen activator
37	ALDH2	P05091	Aldehyde dehydrogenase, mitochondrial
37	CYP17A1	P05093	Steroid 17-alpha-hydroxylase/17,20 lyase
37	LCK	P06239	Tyrosine-protein kinase Lck
37	PGR	P06401	Progesterone receptor
37	FABP1	P07148	Fatty acid-binding protein
37	CTSL	P07711	Procathepsin L
37	CTSB	P07858	Cathepsin B
37	CTSG	P08311	Cathepsin G
37	CYP3A4	P08684	Cytochrome P450 3A4
37	CHRM5	P08912	Muscarinic acetylcholine receptor M5
37	ADRA2A	P08913	Alpha-2A adrenergic receptor
37	CYP2D6	P10635	Cytochrome P450 2D6
37	CYP2C9	P11712	Cytochrome P450 2C9

37	PABPC1	P11940	Polyadenylate-binding protein 1
37	ADRB3	P13945	Beta-3 adrenergic receptor
37	MIF	P14174	Macrophage migration inhibitory factor
37	GABRB2	P47870	Gamma-aminobutyric acid receptor subunit beta-2
37	GABRG2	P18507	Gamma-aminobutyric acid receptor subunit gamma-2
37	PRKCA	P17252	Protein kinase C alpha type
37	FLT1	P17948	Vascular endothelial growth factor receptor 1
37	ADRA2B	P18089	Alpha-2B adrenergic receptor
37	ADRA2C	P18825	Alpha-2C adrenergic receptor
37	CYP11B2	P19099	Cytochrome P450 11B2, mitochondrial
37	TNFRSF1A	P19438	Tumor necrosis factor receptor superfamily member 1A
37	TACR2	P21452	Substance-K receptor
37	DRD1	P21728	D(1A) dopamine receptor
37	ACHE	P22303	Acetylcholinesterase
37	KCNA5	P22460	Potassium voltage-gated channel subfamily A member 5
37	CMA1	P23946	Chymase
37	TBXAS1	P24557	Thromboxane-A synthase
37	CXCR2	P25025	C-X-C chemokine receptor type 2
37	CTSS	P25774	Cathepsin S
37	GABRB3	P28472	Gamma-aminobutyric acid receptor subunit beta-3
37	GABRA5	P31644	Gamma-aminobutyric acid receptor subunit alpha-5
37	GABRA3	P34903	Gamma-aminobutyric acid receptor subunit alpha-3
37	ADORA2A	P29274	Adenosine receptor A2a
37	ADORA2B	P29275	Adenosine receptor A2b
37	TSPO	P30536	Translocator protein
37	ADORA1	P30542	Adenosine receptor A1
37	ALDH3A1	P30838	Aldehyde dehydrogenase, dimeric NADP-preferring
37	GABRA5	P31644	Gamma-aminobutyric acid receptor subunit alpha-5
37	CYP2C19	P33261	Cytochrome P450 2C19
37	HTR7	P34969	5-hydroxytryptamine receptor 7
37	CRHR1	P34998	Corticotropin-releasing factor receptor 1
37	DRD3	P35462	D(3) dopamine receptor
37	OPRD1	P41143	Delta-type opioid receptor
37	GRM5	P41594	Metabotropic glutamate receptor 5

37	HTR2B	P41595	5-hydroxytryptamine receptor 2B
37	CASP3	P42574	Caspase-3
37	CTSK	P43235	Cathepsin K
37	MAPK8	P45983	Mitogen-activated protein kinase 8
37	MAPK9	P45984	Mitogen-activated protein kinase 9
37	MTNR1A	P48039	Melatonin receptor type 1A
37	PREP	P48147	Prolyl endopeptidase
37	MTNR1B	P49286	Melatonin receptor type 1B
37	FNTA	P49354	Protein farnesyltransferase/geranylgeranyltransferase type-1 subunit alpha
37	FNTB	P49356	Protein farnesyltransferase subunit beta
37	CLK3	P49761	Dual specificity protein kinase CLK3
37	RGS4	P49798	Regulator of G-protein signaling 4
37	HTR6	P50406	5-hydroxytryptamine receptor 6
37	PGGT1B	P53609	Geranylgeranyl transferase type-1 subunit beta
37	MAPK10	P53779	Mitogen-activated protein kinase 10
37	VCP	P55072	Transitional endoplasmic reticulum ATPase
37	CASP7	P55210	Caspase-7
37	RGS8	P57771	Regulator of G-protein signaling 8
37	PPP1CA	P62136	Serine/threonine-protein phosphatase PP1-alpha catalytic subunit
37	FKBP1A	P62942	Peptidyl-prolyl cis-trans isomerase FKBP1A
37	ADAM17	P78536	Disintegrin and metalloproteinase domain-containing protein 17
37	PDE4B	Q07343	cAMP-specific 3',5'-cyclic phosphodiesterase 4B
37	TRPC3	Q13507	Short transient receptor potential channel 3
37	GRM2	Q14416	Metabotropic glutamate receptor 2
37	RPS6KA2	Q15349	Ribosomal protein S6 kinase alpha-2 (S6K-alpha-2)
37	MAPK11	Q15759	Mitogen-activated protein kinase 11
37	MAPK14	Q16539	Mitogen-activated protein kinase 14
37	TRPM8	Q7Z2W7	Transient receptor potential cation channel subfamily M member 8
37	S1PR3	Q99500	Sphingosine 1-phosphate receptor 3
37	MGLL	Q99685	Monoglyceride lipase
37	CTRC	Q99895	Chymotrypsin-C
37	TNKS2	Q9H2K2	Poly [ADP-ribose] polymerase tankyrase-2
37	TRPC6	Q9Y210	Short transient receptor potential channel 6
37	PDE10A	Q9Y233	cAMP and cAMP-inhibited cGMP 3',5'-cyclic phosphodiesterase 10A
37	GPR55	Q9Y2T6	G-protein coupled receptor 55

37	DYRK1B	Q9Y463	Dual specificity tyrosine-phosphorylation-regulated kinase 1B
37	PLA2G4B	P0C869	Cytosolic phospholipase A2 beta
37	TLR1	Q15399	Toll-like receptor 1
37	ACER2	Q5QJU3	Alkaline ceramidase 2
37	PIN4	Q9Y237	Peptidyl-prolyl cis-trans isomerase NIMA-interacting 4
37	POLM	Q9NP87	DNA-directed DNA/RNA polymerase mu
37	PRTN3	P24158	Myeloblastin
37	PAM	P19021	Peptidyl-glycine alpha-amidating monooxygenase
37	CHRM5	P08912	Muscarinic acetylcholine receptor M5
37	GABRB1	P18505	Gamma-aminobutyric acid receptor subunit beta-1
37	MPEG1	Q2M385	Macrophage-expressed gene 1 protein
37	CES2	O00748	Cocaine esterase
37	CHRM4	P08173	Muscarinic acetylcholine receptor M4
37	MDH2	P40926	Malate dehydrogenase, mitochondrial
37	CNR2	P34972	Cannabinoid receptor 2
37	FAAH	O00519	Fatty-acid amide hydrolase 1
37	ACR	P10323	Acrosin
37	PLA2G2D	Q9UNK4	Group IID secretory phospholipase A2
37	GSTM1	P09488	Glutathione S-transferase Mu 1
37	PARP10	Q53GL7	Protein mono-ADP-ribosyltransferase PARP10
37	GRM6	O15303	Metabotropic glutamate receptor 6
37	ATF1	P18846	Cyclic AMP-dependent transcription factor ATF-1
37	ALOX5	P09917	Arachidonate 5-lipoxygenase
37	RARB	P10826	Retinoic acid receptor beta
37	THRA	P10827	Thyroid hormone receptor alpha
37	NPC1	O15118	NPC intracellular cholesterol transporter 1
37	CES1	P23141	Liver carboxylesterase 1
37	THRB	P10828	Thyroid hormone receptor beta
37	NAAA	Q02083	N-acylethanolamine-hydrolyzing acid amidase
37	OXER1	Q8TDS5	Oxoeicosanoid receptor 1
37	CTSH	P09668	Pro-cathepsin H
37	LTA4H	P09960	Leukotriene A-4 hydrolase
37	POLH	Q9Y253	DNA polymerase eta
37	XPA	P23025	DNA repair protein complementing XP-A cells
37	CDC25A	P30304	M-phase inducer phosphatase 1

37	NLRP1	Q53GL7	NACHT, LRR and PYD domains-containing protein 1
37	CACNA1D	Q01668	Voltage-dependent L-type calcium channel subunit alpha-1D
37	GABRB2	P47870	Gamma-aminobutyric acid receptor subunit beta-2
37	STS	P08842	Steryl-sulfatase
37	NCEH1	Q6PIU2	Neutral cholesterol ester hydrolase 1
37	ASAH1	Q13510	Acid ceramidase
37	KYNU	Q16719	Kynureninase
37	PPARG	P37231	Peroxisome proliferator-activated receptor gamma
37	CAPN2	P17655	Calpain-2 catalytic subunit
37	P2RY10	O00398	Putative P2Y purinoceptor 10
37	TRPM2	O94759	Transient receptor potential cation channel subfamily M member 2
37	STAT1	P42224	Signal transducer and activator of transcription 1-alpha/beta
37	CYP1A2	P05177	Cytochrome P450 1A2
37	POLK	Q9UBT6	DNA polymerase kappa
37	PPARA	Q07869	Peroxisome proliferator-activated receptor alpha
37	MDH1	P40925	Malate dehydrogenase, cytoplasmic
37	S1PR5	Q9H228	Sphingosine 1-phosphate receptor 5
37	NFKB1	P19838	Nuclear factor NF-kappa-B p105 subunit
37	CNR1	P21554	Cannabinoid receptor 1
37	CDC25C	P30307	M-phase inducer phosphatase 3
37	CYP2C19	P33261	Cytochrome P450 2C19
37	RARA	P10276	Retinoic acid receptor alpha
37	CTRB1	P17538	Chymotrypsinogen B
37	TAOK3	Q9H2K8	Serine/threonine-protein kinase TAO3
37	PHLPP2	Q6ZVD8	PH domain leucine-rich repeat-containing protein phosphatase 2
37	HRH2	P25021	Histamine H2 receptor
37	KAT2A	Q92830	Histone acetyltransferase KAT2A
37	CYP2C9	P11712	Cytochrome P450 2C9
37	RASGRP1	O95267	RAS guanyl-releasing protein 1
37	PTPN6	P29350	Tyrosine-protein phosphatase non-receptor type 6
37	SCN8A	Q9UQD0	Sodium channel protein type 8 subunit alpha
37	PTGES	O14684	Prostaglandin E synthase
37	CDC25B	P30305	M-phase inducer phosphatase 2
37	POLL	Q9UGP5	DNA polymerase lambda

37	SENP7	Q9BQF6	Sentrin-specific protease 7
37	PLA2G2A	P14555	Phospholipase A2, membrane associated
37	AKR1C2	P52895	Aldo-keto reductase family 1 member C2
37	CA9	Q16790	Carbonic anhydrase 9
37	MIF	P14174	Macrophage migration inhibitory factor
37	CA14	Q9ULX7	Carbonic anhydrase 14
37	NR1D1	P20393	Nuclear receptor subfamily 1 group D member 1
37	CTSK	P43235	Cathepsin K
37	CA7	P43166	Carbonic anhydrase 7
37	NCOA1	Q15788	Nuclear receptor coactivator 1
37	S1PR4	O95977	Sphingosine 1-phosphate receptor 4
37	MTNR1A	P48039	Melatonin receptor type 1A
37	HSD17B3	P37058	Testosterone 17-beta-dehydrogenase 3
37	UBE2I	P63279	SUMO-conjugating enzyme UBC9
37	ELANE	P08246	Neutrophil elastase
37	PHOSPHO1	Q8TCT1	Phosphoethanolamine/phosphocholine phosphatase
37	AKR1C1	Q04828	Aldo-keto reductase family 1 member C1
37	HTR4	Q13639	5-hydroxytryptamine receptor 4
37	SLC6A5	Q9Y345	Sodium- and chloride-dependent glycine transporter 2
37	CA12	O43570	Carbonic anhydrase 12
37	TAS1R1	Q7RTX1	Taste receptor type 1 member 1
37	EPHX1	P07099	Epoxide hydrolase 1
37	MMP16	P51512	Matrix metalloproteinase-16
37	LOXL2	Q9Y4K0	Lysyl oxidase homolog 2
37	XDH	P47989	Xanthine dehydrogenase/oxidase
37	NT5E	P21589	5'-nucleotidase
37	HRH1	P35367	Histamine H1 receptor
37	ICMT	O60725	Protein-S-isoprenylcysteine O-methyltransferase
37	SCN3A	Q9NY46	Sodium channel protein type 3 subunit alpha
37	GRK6	P43250	G protein-coupled receptor kinase 6
37	CTSG	P08311	Cathepsin G
39	PTGS1	P23219	Prostaglandin G/H synthase 1
39	PTGS2	P35354	Prostaglandin G/H synthase 2
39	RXRA	P19793	Retinoic acid receptor RXR-alpha
39	NCOA2	Q15596	Nuclear receptor coactivator 2
39	FABP3	P05413	Fatty acid binding protein muscle
39	FABP4	P15090	Fatty acid binding protein adipocyte
39	FFAR1	O14842	Free fatty acid receptor 1
39	CNR1	P21554	Cannabinoid receptor 1 (by homology)

39	HSD11B1	P28845	11-beta-hydroxysteroid dehydrogenase 1
39	PPARG	P37231	Peroxisome proliferator-activated receptor gamma
39	CDC7	O00311	CDC7/DBF4 (Cell division cycle 7-related protein kinase/Activator of S phase kinase)
39	FAAH	O00519	Anandamide amidohydrolase
39	CES2	O00748	Carboxylesterase 2
39	SCD	O00767	Acyl-CoA desaturase
39	PTGES	O14684	Prostaglandin E synthase
39	TERT	O14746	Telomerase reverse transcriptase
39	CYP26A1	O43174	Cytochrome P450 26A1
39	JAK2	O60674	Tyrosine-protein kinase JAK2
39	ROCK2	O75116	Rho-associated protein kinase 2
39	LYPLA1	O75608	Acyl-protein thioesterase 1
39	TNKS	O95271	Tankyrase-1
39	LYPLA2	O95372	Acyl-protein thioesterase 2
39	ABL1	P00519	Tyrosine-protein kinase ABL
39	CYP17A1	P05093	Cytochrome P450 17A1
39	LCK	P06239	Tyrosine-protein kinase LCK
39	EPHX1	P07099	Epoxide hydrolase 1
39	FABP1	P07148	Fatty acid-binding protein, liver
39	CSF1R	P07333	Macrophage colony stimulating factor receptor
39	YES1	P07947	Tyrosine-protein kinase YES
39	MET	P08581	Hepatocyte growth factor receptor
39	ADRA2A	P08913	Alpha-2a adrenergic receptor
39	ALOX5	P09917	Arachidonate 5-lipoxygenase
39	AR	P10275	Androgen Receptor
39	RARA	P10276	Retinoic acid receptor alpha
39	BCL2	P10415	Apoptosis regulator Bcl-2
39	PTPRF	P10586	Receptor-type tyrosine-protein phosphatase F (LAR)
39	KIT	P10721	Stem cell growth factor receptor
39	TOP2A	P11388	DNA topoisomerase II alpha
39	CYP19A1	P11511	Cytochrome P450 19A1
39	IMPDH2	P12268	Inosine-5'-monophosphate dehydrogenase 2
39	SRC	P12931	Tyrosine-protein kinase SRC
39	CYP11B1	P15538	Cytochrome P450 11B1
39	PTPN1	P18031	Protein-tyrosine phosphatase 1B
39	ADRA2C	P18825	Adrenergic receptor alpha-2
39	CYP11B2	P19099	Cytochrome P450 11B2
39	SLC9A1	P19634	Sodium/hydrogen exchanger 1
39	ALOX5AP	P20292	5-lipoxygenase activating protein

39	TBXAS1	P24557	Thromboxane-A synthase
39	MAOB	P27338	Monoamine oxidase B
39	HTR2A	P28223	Serotonin 2a (5-HT2a) receptor (by homology)
39	HTR2C	P28335	Serotonin 2c (5-HT2c) receptor
39	GABRB3	P28472	Gamma-aminobutyric acid receptor subunit beta-3
39	GABRG2	P18507	Gamma-aminobutyric acid receptor subunit gamma-2
39	GABRA1	P14867	Gamma-aminobutyric acid receptor subunit alpha-1
39	GABRA5	P31644	Gamma-aminobutyric acid receptor subunit alpha-5
39	GABRA3	P34903	**Gamma-aminobutyric acid receptor subunit alpha-3**
39	CD38	P28907	Lymphocyte differentiation antigen CD38
39	CNR2	P34972	Cannabinoid receptor 2
39	CRHR1	P34998	Corticotropin releasing factor receptor 1
39	NOS2	P35228	Nitric oxide synthase, inducible (by homology)
39	OPRM1	P35372	Mu opioid receptor
39	GCK	P35557	Hexokinase type IV
39	KDR	P35968	Vascular endothelial growth factor receptor 2
39	CHRNA7	P36544	Neuronal acetylcholine receptor protein alpha-7 subunit
39	HSD17B2	P37059	Estradiol 17-beta-dehydrogenase 2
39	OPRD1	P41143	Delta opioid receptor
39	CASR	P41180	Calcium sensing receptor
39	CTSK	P43235	Cathepsin K
39	CHRNA4	P43681	Neuronal acetylcholine receptor subunit alpha-4
39	GABRA2	P47869	Gamma-aminobutyric acid receptor subunit alpha-2
39	CHRNB2	P17787	Neuronal acetylcholine receptor subunit beta-2
39	GCGR	P47871	Glucagon receptor
39	MTNR1A	P48039	Melatonin receptor 1A
39	SLC6A9	P48067	Glycine transporter 1 (by homology)
39	CSNK1D	P48730	Casein kinase I delta
39	MTNR1B	P49286	Melatonin receptor 1B
39	PSEN2	P49810	Gamma-secretase
39	PSENEN	Q9NZ42	Gamma-secretase subunit PEN-2
39	NCSTN	Q92542	Nicastrin

39	APH1A	Q96BI3	Gamma-secretase subunit APH-1A
39	PSEN1	P49768	Presenilin-1
39	APH1B	Q8WW43	Gamma-secretase subunit APH-1B
39	HTR6	P50406	Serotonin 6 (5-HT6) receptor
39	RORC	P51449	Nuclear receptor ROR-gamma
39	KIF11	P52732	Kinesin-like protein 1
39	LIMK2	P53671	LIM domain kinase 2
39	CACNA2D1	P54289	Voltage-gated calcium channel alpha2/delta subunit 1
39	EPHB4	P54760	Ephrin receptor
39	BRPF1	P55201	Peregrin
39	FABP5	Q01469	Fatty acid binding protein epidermal
39	PPARD	Q03181	Peroxisome proliferator-activated receptor delta
39	GRIN1	Q05586	Glutamate receptor ionotropic, NMDA 1
39	GRIN2B	Q13224	Glutamate receptor ionotropic, NMDA 2B
39	MCL1	Q07820	Induced myeloid leukemia cell differentiation protein Mcl-1
39	PPARA	Q07869	Peroxisome proliferator-activated receptor alpha
39	NR1H3	Q13133	LXR-alpha
39	GRIN2B	Q13224	Glutamate [NMDA] receptor subunit epsilon 2
39	ASAH1	Q13510	Acid ceramidase
39	NPY5R	Q15761	Neuropeptide Y receptor type 5
39	SCN9A	Q15858	Sodium channel protein type IX alpha subunit
39	MAPK14	Q16539	MAP kinase p38 alpha
39	CALCRL	Q16602	Calcitonin gene-related peptide type 1 receptor
39	RAMP1	O60894	Receptor activity-modifying protein 1
39	QPCT	Q16769	Glutaminyl-peptide cyclotransferase
39	PFKFB3	Q16875	6-phosphofructo-2-kinase/fructose-2,6-bisphosphatase 3
39	TRPV1	Q8NER1	Vanilloid receptor
39	GPR119	Q8TDV5	Glucose-dependent insulinotropic receptor
39	AURKB	Q96GD4	Serine/threonine-protein kinase Aurora-B
39	P2RX7	Q99572	P2X purinoceptor 7 (by homology)
39	HRH4	Q9H3N8	Histamine H4 receptor
39	HRH3	Q9Y5N1	Histamine H3 receptor
39	LPAR3	Q9UBY5	Lysophosphatidic acid receptor 3
39	P2RY10	O00398	Putative P2Y purinoceptor 10
39	LPAR4	Q99677	Lysophosphatidic acid receptor 4
39	TLR2	O60603	Toll-like receptor 2

39	GPR174	Q9BXC1	Probable G-protein coupled receptor 174
39	LPAR6	P43657	Lysophosphatidic acid receptor 6
39	LPAR1	Q92633	Lysophosphatidic acid receptor 1
39	GPR34	Q9UPC5	Probable G-protein coupled receptor 34
39	OXER1	Q8TDS5	Oxoeicosanoid receptor 1
39	LPAR2	Q9HBW0	Lysophosphatidic acid receptor 2
39	POLM	Q9NP87	DNA-directed DNA/RNA polymerase mu
39	SLC25A20	O43772	Mitochondrial carnitine/acylcarnitine carrier protein
39	POLH	Q9Y253	DNA polymerase eta
39	ACER2	Q5QJU3	Alkaline ceramidase 2
39	SPTLC2	O15270	Serine palmitoyltransferase 2
39	PLA2G2C	Q5R387	Putative inactive group IIC secretory phospholipase A2
39	CERT1	Q9Y5P4	Ceramide transfer protein
39	ADH7	P40394	All-trans-retinol dehydrogenase [NAD(+)] ADH7
39	PAFAH1B2	P68402	Platelet-activating factor acetylhydrolase IB subunit beta
39	SPTLC1	O15269	Serine palmitoyltransferase 1
39	POLK	Q9UBT6	DNA polymerase kappa
39	PLA2G5	P39877	Calcium-dependent phospholipase A2
39	SELP	P16109	P-selectin
39	PAM	P19021	Peptidyl-glycine alpha-amidating monooxygenase
39	PDCD4	Q53EL6	Programmed cell death protein 4
39	MPEG1	Q2M385	Macrophage-expressed gene 1 protein
39	ENPP2	Q13822	Ectonucleotide pyrophosphatase/phosphodiesterase family member 2
39	PRKCA	P17252	Protein kinase C alpha type
39	SMPD2	O60906	Sphingomyelin phosphodiesterase 2
39	KAT5	Q92993	Histone acetyltransferase KAT5
39	PLA2G10	O15496	Group 10 secretory phospholipase A2
39	CES1	P23141	Liver carboxylesterase 1
39	EPHX2	P34913	Bifunctional epoxide hydrolase 2
39	NDUFAB1	O14561	Acyl carrier protein, mitochondrial
39	NDUFAF1	Q9Y375	Complex I intermediate-associated protein 30, mitochondrial
39	NDUFA1	O15239	NADH dehydrogenase [ubiquinone] 1 alpha subcomplex subunit 1
39	NDUFA2	O43678	NADH dehydrogenase [ubiquinone] 1 alpha subcomplex subunit 2

39	NDUFA3	O95167	NADH dehydrogenase [ubiquinone] 1 alpha subcomplex subunit 3
39	NDUFA6	P56556	NADH dehydrogenase [ubiquinone] 1 alpha subcomplex subunit 6
39	NDUFA7	O95182	NADH dehydrogenase [ubiquinone] 1 alpha subcomplex subunit 7
39	NDUFA5	Q16718	NADH dehydrogenase [ubiquinone] 1 alpha subcomplex subunit 5
39	NDUFA8	P51970	NADH dehydrogenase [ubiquinone] 1 alpha subcomplex subunit 8
39	NDUFA9	Q16795	NADH dehydrogenase [ubiquinone] 1 alpha subcomplex subunit 9, mitochondrial
39	NDUFA10	O95299	NADH dehydrogenase [ubiquinone] 1 alpha subcomplex subunit 10, mitochondrial
39	NDUFA11	Q86Y39	NADH dehydrogenase [ubiquinone] 1 alpha subcomplex subunit 11
39	NDUFA12	Q9UI09	NADH dehydrogenase [ubiquinone] 1 alpha subcomplex subunit 12
39	NDUFA13	Q9P0J0	NADH dehydrogenase [ubiquinone] 1 alpha subcomplex subunit 13
39	NDUFB2	O95178	NADH dehydrogenase [ubiquinone] 1 beta subcomplex subunit 2, mitochondrial
39	NDUFB1	O75438	NADH dehydrogenase [ubiquinone] 1 beta subcomplex subunit 1
39	NDUFB3	O43676	NADH dehydrogenase [ubiquinone] 1 beta subcomplex subunit 3
39	NDUFB4	O95168	NADH dehydrogenase [ubiquinone] 1 beta subcomplex subunit 4
39	NDUFB5	O43674	NADH dehydrogenase [ubiquinone] 1 beta subcomplex subunit 5, mitochondrial
39	NDUFB6	O95139	NADH dehydrogenase [ubiquinone] 1 beta subcomplex subunit 6
39	NDUFB7	P17568	NADH dehydrogenase [ubiquinone] 1 beta subcomplex subunit 7
39	NDUFB8	O95169	NADH dehydrogenase [ubiquinone] 1 beta subcomplex subunit 8, mitochondrial
39	NDUFB9	Q9Y6M9	NADH dehydrogenase [ubiquinone] 1 beta subcomplex subunit 9
39	NDUFB10	O96000	NADH dehydrogenase [ubiquinone] 1 beta subcomplex subunit 10
39	NDUFB11	Q9NX14	NADH dehydrogenase [ubiquinone] 1 beta subcomplex subunit 11, mitochondrial
39	NDUFC1	O43677	NADH dehydrogenase [ubiquinone] 1 subunit C1, mitochondrial

39	NDUFC2	O95298	NADH dehydrogenase [ubiquinone] 1 subunit C2
39	NDUFAF2	Q8N183	NADH dehydrogenase [ubiquinone] 1 alpha subcomplex assembly factor 2
39	NDUFAF4	Q9P032	NADH dehydrogenase [ubiquinone] 1 alpha subcomplex assembly factor 4
39	NDUFAF3	Q9BU61	NADH dehydrogenase [ubiquinone] 1 alpha subcomplex assembly factor 3
39	NDUFS1	P28331	NADH-ubiquinone oxidoreductase 75 kDa subunit, mitochondrial
39	NDUFS2	O75306	NADH dehydrogenase [ubiquinone] iron-sulfur protein 2, mitochondrial
39	NDUFS3	O75489	NADH dehydrogenase [ubiquinone] iron-sulfur protein 3, mitochondrial
39	NDUFS4	O43181	NADH dehydrogenase [ubiquinone] iron-sulfur protein 4, mitochondrial
39	NDUFS5	O43920	NADH dehydrogenase [ubiquinone] iron-sulfur protein 5
39	NDUFS6	O75380	NADH dehydrogenase [ubiquinone] iron-sulfur protein 6, mitochondrial
39	NDUFS7	O75251	NADH dehydrogenase [ubiquinone] iron-sulfur protein 7, mitochondrial
39	NDUFS8	O00217	NADH dehydrogenase [ubiquinone] iron-sulfur protein 8, mitochondrial
39	NDUFV1	P49821	NADH dehydrogenase [ubiquinone] flavoprotein 1, mitochondrial
39	NDUFV2	P19404	NADH dehydrogenase [ubiquinone] flavoprotein 2, mitochondrial
39	NDUFV3	P56181	NADH dehydrogenase [ubiquinone] flavoprotein 3, mitochondrial
39	MT-ND2	P03891	NADH-ubiquinone oxidoreductase chain 2
39	MT-ND1	P03886	NADH-ubiquinone oxidoreductase chain 1
39	MT-ND3	P03897	NADH-ubiquinone oxidoreductase chain 3
39	MT-ND4L	P03901	NADH-ubiquinone oxidoreductase chain 4L
39	MT-ND5	P03915	NADH-ubiquinone oxidoreductase chain 5
39	MT-ND6	P03923	NADH-ubiquinone oxidoreductase chain 6
39	NDUFA4L2	Q9NRX3	NADH dehydrogenase [ubiquinone] 1 alpha subcomplex subunit 4-like 2
39	SLCO2A1	Q92959	Solute carrier organic anion transporter family member 2A1
39	POLB	P06746	DNA polymerase beta
39	POLA1	P09884	DNA polymerase alpha catalytic subunit
39	LY96	Q9Y6Y9	Lymphocyte antigen 96

39	NDUFA4	O00483	Cytochrome c oxidase subunit NDUFA4
39	MT-ND4	P03905	NADH-ubiquinone oxidoreductase chain 4
39	IARS1	P41252	Isoleucine--tRNA ligase, cytoplasmic
39	PLA2G2A	P14555	Phospholipase A2, membrane associated
39	GPR18	Q14330	N-arachidonyl glycine receptor
39	NAAA	Q02083	N-acylethanolamine-hydrolyzing acid amidase
39	DNM1	Q05193	Dynamin-1
39	POLL	Q9UGP5	DNA polymerase lambda
39	DAGLA	Q9Y4D2	Sn1-specific diacylglycerol lipase alpha
39	NOD1	Q9Y239	Nucleotide-binding oligomerization domain-containing protein 1
39	ADH1B	P00325	All-trans-retinol dehydrogenase [NAD(+)] ADH1B
39	KAT2B	Q92831	Histone acetyltransferase KAT2B
39	HMGCR	P04035	3-hydroxy-3-methylglutaryl-coenzyme A reductase
39	PLA2G4B	P0C869	Cytosolic phospholipase A2 beta
39	PRKCE	Q02156	Protein kinase C epsilon type
39	CDC25B	P30305	M-phase inducer phosphatase 2
39	KDM5A	P29375	Lysine-specific demethylase 5A
39	SLC22A8	Q8TCC7	Solute carrier family 22 member 8
39	HSD17B3	P37058	Testosterone 17-beta-dehydrogenase 3
39	TLR4	O00206	Toll-like receptor 4
39	PTPN13	Q12923	Tyrosine-protein phosphatase non-receptor type 13
39	APEX1	P27695	DNA-(apurinic or apyrimidinic site) lyase
39	SLC22A6	Q4U2R8	Solute carrier family 22 member 6
39	SPHK1	Q9NYA1	Sphingosine kinase 1
39	LPAR5	Q9H1C0	Lysophosphatidic acid receptor 5
39	ADH1A	P07327	Alcohol dehydrogenase 1A
40	PTGS1	P23219	Prostaglandin G/H synthase 1
40	PTGS2	P35354	Prostaglandin G/H synthase 2
40	RXRA	P19793	Retinoic acid receptor RXR-alpha
40	NCOA2	Q15596	Nuclear receptor coactivator 2
40	SLC6A2	P23975	Sodium-dependent noradrenaline transporter
40	FABP3	P05413	Fatty acid binding protein muscle
40	FABP4	P15090	Fatty acid binding protein adipocyte
40	CNR1	P21554	Cannabinoid receptor 1 (by homology)
40	HSD11B1	P28845	11-beta-hydroxysteroid dehydrogenase 1
40	PPARG	P37231	Peroxisome proliferator-activated receptor gamma
40	ABL1	P00519	Tyrosine-protein kinase ABL

40	ALOX5	P09917	Arachidonate 5-lipoxygenase
40	ALOX5AP	P20292	5-lipoxygenase activating protein
40	AOC3	Q16853	Amine oxidase, copper containing
40	AR	P10275	Androgen Receptor
40	BRPF1	P55201	Peregrin
40	CALCRL	Q16602	Calcitonin gene-related peptide type 1 receptor
40	RAMP1	O60894	Receptor activity-modifying protein 1
40	CD38	P28907	Lymphocyte differentiation antigen CD38
40	CDC7	O00311	CDC7/DBF4 (Cell division cycle 7-related protein kinase/Activator of S phase kinase)
40	CES2	O00748	Carboxylesterase 2
40	CHRNA4	P43681	Neuronal acetylcholine receptor subunit alpha-4
40	CHRNB2	P17787	Neuronal acetylcholine receptor subunit beta-2
40	CHRNA7	P36544	Neuronal acetylcholine receptor protein alpha-7 subunit
40	CHUK	O15111	Inhibitor of NF-kappa-B kinase (IKK)
40	CNR2	P34972	Cannabinoid receptor 2
40	CRHR1	P34998	Corticotropin releasing factor receptor 1
40	CSF1R	P07333	Macrophage colony stimulating factor receptor
40	CYP11B1	P15538	Cytochrome P450 11B1
40	CYP11B2	P19099	Cytochrome P450 11B2
40	CYP17A1	P05093	Cytochrome P450 17A1
40	CYP19A1	P11511	Cytochrome P450 19A1
40	EPHB4	P54760	Ephrin receptor
40	F2R	P25116	Proteinase-activated receptor 1
40	FAAH	O00519	Anandamide amidohydrolase
40	FABP1	P07148	Fatty acid-binding protein, liver
40	FABP5	Q01469	Fatty acid binding protein epidermal
40	FFAR1	O14842	Free fatty acid receptor 1
40	FLT1	P17948	Vascular endothelial growth factor receptor 1
40	GABRA2	P47869	Gamma-aminobutyric acid receptor subunit alpha-2
40	GABRB3	P28472	Gamma-aminobutyric acid receptor subunit beta-3
40	GABRG2	P18507	Gamma-aminobutyric acid receptor subunit gamma-2
40	GABRA3	P34903	Gamma-aminobutyric acid receptor subunit alpha-3
40	GABRA1	P14867	Gamma-aminobutyric acid receptor subunit

			alpha-1
40	GABRA5	P31644	Gamma-aminobutyric acid receptor subunit alpha-5
40	GCGR	P47871	Glucagon receptor
40	GPR119	Q8TDV5	Glucose-dependent insulinotropic receptor
40	GRIA2	P42262	Glutamate receptor ionotropic, AMPA 2
40	GRIN2B	Q13224	Glutamate [NMDA] receptor subunit epsilon 2
40	GRM5	P41594	Metabotropic glutamate receptor 5
40	GSK3A	P49840	Glycogen synthase kinase-3 alpha
40	HCRTR1	O43613	Orexin receptor 1
40	HCRTR2	O43614	Orexin receptor 2
40	HIPK1	Q86Z02	Homeodomain-interacting protein kinase 1
40	HRH3	Q9Y5N1	Histamine H3 receptor
40	HRH4	Q9H3N8	Histamine H4 receptor
40	HTR2A	P28223	Serotonin 2a (5-HT2a) receptor (by homology)
40	HTR2B	P41595	Serotonin 2b (5-HT2b) receptor
40	HTR2C	P28335	Serotonin 2c (5-HT2c) receptor
40	HTR6	P50406	Serotonin 6 (5-HT6) receptor
40	ICMT	O60725	Isoprenylcysteine carboxyl methyltransferase
40	IMPDH2	P12268	Inosine-5'-monophosphate dehydrogenase 2
40	KDR	P35968	Vascular endothelial growth factor receptor 2
40	KIF11	P52732	Kinesin-like protein 1
40	KIT	P10721	Stem cell growth factor receptor
40	LCK	P06239	Tyrosine-protein kinase LCK
40	LIMK2	P53671	LIM domain kinase 2
40	MAOB	P27338	Monoamine oxidase B
40	MAPK1	P28482	MAP kinase ERK2
40	MAPK14	Q16539	MAP kinase p38 alpha
40	MCL1	Q07820	Induced myeloid leukemia cell differentiation protein Mcl-1
40	MET	P08581	Hepatocyte growth factor receptor
40	MTNR1A	P48039	Melatonin receptor 1A
40	MTNR1B	P49286	Melatonin receptor 1B
40	MTOR	P42345	Serine/threonine-protein kinase mTOR
40	NAMPT	P43490	Nicotinamide phosphoribosyltransferase
40	NOS2	P35228	Nitric oxide synthase, inducible (by homology)
40	NPY5R	Q15761	Neuropeptide Y receptor type 5
40	NR1H3	Q13133	LXR-alpha
40	NTRK1	P04629	Nerve growth factor receptor Trk-A
40	OPRD1	P41143	Delta opioid receptor

40	PDE4D	Q08499	Phosphodiesterase 4D
40	PDE5A	O76074	Phosphodiesterase 5A
40	PDE6D	O43924	Phosphodiesterase 6D
40	PDGFRB	P09619	Platelet-derived growth factor receptor beta
40	PFKFB3	Q16875	6-phosphofructo-2-kinase/fructose-2,6-bisphosphatase 3
40	PGGT1B	P53609	Geranylgeranyl transferase type-1 subunit beta
40	FNTA	P49354	Protein farnesyltransferase/geranylgeranyltransferase type-1 subunit alpha
40	PIM1	P11309	Serine/threonine-protein kinase PIM1
40	PORCN	Q9H237	Probable protein-cysteine N-palmitoyltransferase porcupine
40	PPARA	Q07869	Peroxisome proliferator-activated receptor alpha
40	PPARD	Q03181	Peroxisome proliferator-activated receptor delta
40	PSEN2	P49810	Gamma-secretase
40	PSENEN	Q9NZ42	Gamma-secretase subunit PEN-2
40	NCSTN	Q92542	Nicastrin
40	APH1A	Q96BI3	Gamma-secretase subunit APH-1A
40	PSEN1	P49768	Presenilin-1
40	APH1B	Q8WW43	Gamma-secretase subunit APH-1B
40	PTGES	O14684	Prostaglandin E synthase
40	PTPN1	P18031	Protein-tyrosine phosphatase 1B
40	PTPN2	P17706	T-cell protein-tyrosine phosphatase
40	QPCT	Q16769	Glutaminyl-peptide cyclotransferase
40	RARA	P10276	Retinoic acid receptor alpha
40	SCD	O00767	Acyl-CoA desaturase
40	SCN9A	Q15858	Sodium channel protein type IX alpha subunit
40	SLC6A9	P48067	Glycine transporter 1 (by homology)
40	SLC9A1	P19634	Sodium/hydrogen exchanger 1
40	SRC	P12931	Tyrosine-protein kinase SRC
40	TERT	O14746	Telomerase reverse transcriptase
40	TNKS	O95271	Tankyrase-1
40	TRPV1	Q8NER1	Vanilloid receptor
40	VCP	P55072	Transitional endoplasmic reticulum ATPase
40	YES1	P07947	Tyrosine-protein kinase YES
40	P2RY10	O00398	Putative P2Y purinoceptor 10
40	LPAR4	Q99677	Lysophosphatidic acid receptor 4
40	GPR174	Q9BXC1	Probable G-protein coupled receptor 174
40	LPAR3	Q9UBY5	Lysophosphatidic acid receptor 3

40	POLM	Q9NP87	DNA-directed DNA/RNA polymerase mu
40	OXER1	Q8TDS5	Oxoeicosanoid receptor 1
40	POLH	Q9Y253	DNA polymerase eta
40	LPAR6	P43657	Lysophosphatidic acid receptor 6
40	LPAR2	Q9HBW0	Lysophosphatidic acid receptor 2
40	PAFAH1B2	P68402	Platelet-activating factor acetylhydrolase IB subunit beta
40	GPR34	Q9UPC5	Probable G-protein coupled receptor 34
40	POLK	Q9UBT6	DNA polymerase kappa
40	KAT5	Q92993	Histone acetyltransferase KAT5
40	LPAR1	Q92633	Lysophosphatidic acid receptor 1
40	PAM	P19021	Peptidyl-glycine alpha-amidating monooxygenase
40	SPTLC2	O15270	Serine palmitoyltransferase 2
40	MT-ND1	P03886	NADH-ubiquinone oxidoreductase chain 1
40	MT-ND2	P03891	NADH-ubiquinone oxidoreductase chain 2
40	MT-ND3	P03897	NADH-ubiquinone oxidoreductase chain 3
40	MT-ND4L	P03901	NADH-ubiquinone oxidoreductase chain 4L
40	MT-ND5	P03915	NADH-ubiquinone oxidoreductase chain 5
40	MT-ND6	P03923	NADH-ubiquinone oxidoreductase chain 6
40	NDUFA1	O15239	NADH dehydrogenase [ubiquinone] 1 alpha subcomplex subunit 1
40	NDUFA10	O95299	NADH dehydrogenase [ubiquinone] 1 alpha subcomplex subunit 10, mitochondrial
40	NDUFA11	Q86Y39	NADH dehydrogenase [ubiquinone] 1 alpha subcomplex subunit 11
40	NDUFA12	Q9UI09	NADH dehydrogenase [ubiquinone] 1 alpha subcomplex subunit 12
40	NDUFA13	Q9P0J0	NADH dehydrogenase [ubiquinone] 1 alpha subcomplex subunit 13
40	NDUFA2	O43678	NADH dehydrogenase [ubiquinone] 1 alpha subcomplex subunit 2
40	NDUFA3	O95167	NADH dehydrogenase [ubiquinone] 1 alpha subcomplex subunit 3
40	NDUFA4L2	Q9NRX3	NADH dehydrogenase [ubiquinone] 1 alpha subcomplex subunit 4-like 2
40	NDUFA5	Q16718	NADH dehydrogenase [ubiquinone] 1 alpha subcomplex subunit 5
40	NDUFA6	P56556	NADH dehydrogenase [ubiquinone] 1 alpha subcomplex subunit 6
40	NDUFA7	O95182	NADH dehydrogenase [ubiquinone] 1 alpha subcomplex subunit 7
40	NDUFA8	P51970	NADH dehydrogenase [ubiquinone] 1 alpha

			subcomplex subunit 8
40	NDUFA9	Q16795	NADH dehydrogenase [ubiquinone] 1 alpha subcomplex subunit 9, mitochondrial
40	NDUFAB1	O14561	Acyl carrier protein, mitochondrial
40	NDUFAF1	Q9Y375	Complex I intermediate-associated protein 30, mitochondrial
40	NDUFAF2	Q8N183	NADH dehydrogenase [ubiquinone] 1 alpha subcomplex assembly factor 2
40	NDUFAF3	Q9BU61	NADH dehydrogenase [ubiquinone] 1 alpha subcomplex assembly factor 3
40	NDUFAF4	Q9P032	NADH dehydrogenase [ubiquinone] 1 alpha subcomplex assembly factor 4
40	NDUFB1	O75438	NADH dehydrogenase [ubiquinone] 1 beta subcomplex subunit 1
40	NDUFB10	O96000	NADH dehydrogenase [ubiquinone] 1 beta subcomplex subunit 10
40	NDUFB11	Q9NX14	NADH dehydrogenase [ubiquinone] 1 beta subcomplex subunit 11, mitochondrial
40	NDUFB2	O95178	NADH dehydrogenase [ubiquinone] 1 beta subcomplex subunit 2, mitochondrial
40	NDUFB3	O43676	NADH dehydrogenase [ubiquinone] 1 beta subcomplex subunit 3
40	NDUFB4	O95168	NADH dehydrogenase [ubiquinone] 1 beta subcomplex subunit 4
40	NDUFB5	O43674	NADH dehydrogenase [ubiquinone] 1 beta subcomplex subunit 5, mitochondrial
40	NDUFB6	O95139	NADH dehydrogenase [ubiquinone] 1 beta subcomplex subunit 6
40	NDUFB7	P17568	NADH dehydrogenase [ubiquinone] 1 beta subcomplex subunit 7
40	NDUFB8	O95169	NADH dehydrogenase [ubiquinone] 1 beta subcomplex subunit 8, mitochondrial
40	NDUFB9	Q9Y6M9	NADH dehydrogenase [ubiquinone] 1 beta subcomplex subunit 9
40	NDUFC1	O43677	NADH dehydrogenase [ubiquinone] 1 subunit C1, mitochondrial
40	NDUFC2	O95298	NADH dehydrogenase [ubiquinone] 1 subunit C2
40	NDUFS1	P28331	NADH-ubiquinone oxidoreductase 75 kDa subunit, mitochondrial
40	NDUFS2	O75306	NADH dehydrogenase [ubiquinone] iron-sulfur protein 2, mitochondrial
40	NDUFS3	O75489	NADH dehydrogenase [ubiquinone] iron-

			sulfur protein 3, mitochondrial
40	NDUFS4	O43181	NADH dehydrogenase [ubiquinone] iron-sulfur protein 4, mitochondrial
40	NDUFS5	O43920	NADH dehydrogenase [ubiquinone] iron-sulfur protein 5
40	NDUFS6	O75380	NADH dehydrogenase [ubiquinone] iron-sulfur protein 6, mitochondrial
40	NDUFS7	O75251	NADH dehydrogenase [ubiquinone] iron-sulfur protein 7, mitochondrial
40	NDUFS8	O00217	NADH dehydrogenase [ubiquinone] iron-sulfur protein 8, mitochondrial
40	NDUFV1	P49821	NADH dehydrogenase [ubiquinone] flavoprotein 1, mitochondrial
40	NDUFV2	P19404	NADH dehydrogenase [ubiquinone] flavoprotein 2, mitochondrial
40	NDUFV3	P56181	NADH dehydrogenase [ubiquinone] flavoprotein 3, mitochondrial
40	EPHX2	P34913	Bifunctional epoxide hydrolase 2
40	NDUFA4	O00483	Cytochrome c oxidase subunit NDUFA4
40	TLR2	O60603	Toll-like receptor 2
40	KAT2B	Q92831	Histone acetyltransferase KAT2B
40	IARS1	P41252	Isoleucine--tRNA ligase, cytoplasmic
40	MT-ND4	P03905	NADH-ubiquinone oxidoreductase chain 4
40	GPR18	Q14330	N-arachidonyl glycine receptor
40	POLL	Q9UGP5	DNA polymerase lambda
40	PRKCA	P17252	Protein kinase C alpha type
40	PTPN13	Q12923	Tyrosine-protein phosphatase non-receptor type 13
40	DAGLA	Q9Y4D2	Sn1-specific diacylglycerol lipase alpha
40	POLB	P06746	DNA polymerase beta
40	ENPP2	Q13822	Ectonucleotide pyrophosphatase/phosphodiesterase family member 2
41	AR	P10275	Androgen Receptor
41	AKR1C3	P42330	Aldo-keto-reductase family 1 member C3
41	PSEN2	P49810	Presenilin-2
41	PSENEN	Q9NZ42	Gamma-secretase subunit PEN-2
41	NCSTN	Q92542	Nicastrin
41	APH1A	Q96BI3	Gamma-secretase subunit APH-1A
41	PSEN1	P49768	Presenilin-1
41	APH1B	Q8WW43	Gamma-secretase subunit APH-1B
41	EPHX2	P34913	Epoxide hydratase
41	IDO1	P14902	Indoleamine 2,3-dioxygenase

41	SQLE	Q14534	Squalene monooxygenase
41	SHBG	P04278	Testis-specific androgen-binding protein
41	MAPK14	Q16539	MAP kinase p38 alpha (by homology)
41	HSD17B2	P37059	Estradiol 17-beta-dehydrogenase 2
41	JAK1	P23458	Tyrosine-protein kinase JAK1
41	JAK2	O60674	Tyrosine-protein kinase JAK2
41	GCGR	P47871	Glucagon receptor
41	CCNB3	Q8WWL7	G2/mitotic-specific cyclin-B3
41	CCNB1	P14635	G2/mitotic-specific cyclin-B1
41	CCNB2	O95067	G2/mitotic-specific cyclin-B2
41	GSK3B	P49841	Glycogen synthase kinase-3 beta
41	PRKCG	P05129	Protein kinase C gamma
41	PRKCD	Q05655	Protein kinase C delta
41	PRKCB	P05771	Protein kinase C beta
41	CDK1	P06493	Cyclin-dependent kinase 1
41	PRKCE	Q02156	Protein kinase C epsilon
41	PRKCH	P24723	Protein kinase C eta
41	PRKCQ	Q04759	Protein kinase C theta
41	RASGRP1	O95267	RAS guanyl-releasing protein 1 (by homology)
41	IL6ST	P40189	Interleukin-6 receptor subunit beta
41	MAOA	P21397	Monoamine oxidase A (by homology)
41	MAOB	P27338	Monoamine oxidase B (by homology)
41	KCNH2	Q12809	HERG
41	BACE1	P56817	Beta-secretase 1
41	JAK3	P52333	Tyrosine-protein kinase JAK3
41	TYK2	P29597	Tyrosine-protein kinase TYK2
41	GRM5	P41594	Metabotropic glutamate receptor 5
41	CCR1	P32246	C-C chemokine receptor type 1
41	PER2	O15055	Period circadian protein homolog 2
41	C5AR1	P21730	C5a anaphylatoxin chemotactic receptor
41	TRPA1	O75762	Transient receptor potential cation channel subfamily A member 1
41	F2R	P25116	Proteinase-activated receptor 1
41	PGGT1B	P53609	Geranylgeranyl transferase type I beta subunit
41	NPY5R	Q15761	Neuropeptide Y receptor type 5
41	SLC6A3	Q01959	Dopamine transporter
41	ADORA3	P0DMS8	Adenosine A3 receptor
41	MMP1	P03956	Matrix metalloproteinase 1
41	MAPK3	P27361	MAP kinase ERK1
41	MAP3K5	Q99683	Mitogen-activated protein kinase kinase kinase 5
41	CNR2	P34972	Cannabinoid receptor 2

41	SCN9A	Q15858	Sodium channel protein type IX alpha subunit
41	MAPK8	P45983	c-Jun N-terminal kinase 1
41	MMP3	P08254	Matrix metalloproteinase 3
41	MMP9	P14780	Matrix metalloproteinase 9
41	FDFT1	P37268	Squalene synthetase
41	CCR5	P51681	C-C chemokine receptor type 5
41	CHRM1	P11229	Muscarinic acetylcholine receptor M1
41	CHRM3	P20309	Muscarinic acetylcholine receptor M3
41	ADA	P00813	Adenosine deaminase
41	ABCC9	O60706	Sulfonylurea receptor 2
41	PYGL	P06737	Liver glycogen phosphorylase
41	PDE2A	O00408	Phosphodiesterase 2A
41	PDE10A	Q9Y233	Phosphodiesterase 10A
41	EGFR	P00533	Epidermal growth factor receptor erbB1
41	TYMS	P04818	Thymidylate synthase
41	MAPK1	P28482	MAP kinase ERK2
41	HSD17B3	P37058	Estradiol 17-beta-dehydrogenase 3
41	LTA4H	P09960	Leukotriene A4 hydrolase
41	CYP19A1	P11511	Cytochrome P450 19A1
41	GABBR2	O75899	Gamma-aminobutyric acid type B receptor subunit 2
41	GABBR1	Q9UBS5	Gamma-aminobutyric acid type B receptor subunit 1
41	TTL	Q8NG68	Tubulin--tyrosine ligase
41	UGT2B7	P16662	UDP-glucuronosyltransferase 2B7
41	PRCP	P42785	Lysosomal Pro-X carboxypeptidase
41	GRM2	Q14416	Metabotropic glutamate receptor 2
41	PIM1	P11309	Serine/threonine-protein kinase PIM1
41	PIM3	Q86V86	Serine/threonine-protein kinase PIM3
41	CA2	P00918	Carbonic anhydrase II
41	CA1	P00915	Carbonic anhydrase I
41	CA6	P23280	Carbonic anhydrase VI
41	CA4	P22748	Carbonic anhydrase IV
41	ICMT	O60725	Isoprenylcysteine carboxyl methyltransferase
41	CYP11B1	P15538	Cytochrome P450 11B1
41	CYP11B2	P19099	Cytochrome P450 11B2
41	AVPR1A	P37288	Vasopressin V1a receptor
41	LRRK2	Q5S007	Leucine-rich repeat serine/threonine-protein kinase 2
41	THRB	P10828	Thyroid hormone receptor beta-1
41	KCNA5	P22460	Voltage-gated potassium channel subunit Kv1.5
41	TGFBR1	P36897	TGF-beta receptor type I

41	PIM2	Q9P1W9	Serine/threonine-protein kinase PIM2
41	MDM2	Q00987	p53-binding protein Mdm-2
41	CHRM4	P08173	Muscarinic acetylcholine receptor M4
41	MTNR1A	P48039	Melatonin receptor 1A
41	MTNR1B	P49286	Melatonin receptor 1B
41	CHRM5	P08912	Muscarinic acetylcholine receptor M5
41	TACR2	P21452	Neurokinin 2 receptor
41	TACR1	P25103	Neurokinin 1 receptor
41	CYP2C9	P11712	Cytochrome P450 2C9
41	MAP2K1	Q02750	Dual specificity mitogen-activated protein kinase kinase 1
41	EPHX1	P07099	Epoxide hydrolase 1
41	CYP3A4	P08684	Cytochrome P450 3A4
41	AVPR2	P30518	Vasopressin V2 receptor (by homology)
41	P2RX7	Q99572	P2X purinoceptor 7
41	PRMT3	O60678	Protein arginine N-methyltransferase 3
41	SPHK2	Q9NRA0	Sphingosine kinase 2
41	SPHK1	Q9NYA1	Sphingosine kinase 1
41	DRD2	P14416	Dopamine D2 receptor
41	DRD4	P21917	Dopamine D4 receptor
42	GABRQ	Q9UN88	Gamma-aminobutyric acid receptor subunit theta
42	BBOX1	O75936	Gamma-butyrobetaine dioxygenase
42	SLCO2A1	Q92959	Solute carrier organic anion transporter family member 2A1
42	SLC6A11	P48066	Sodium- and chloride-dependent GABA transporter 3
42	PHF8	Q9UPP1	Histone lysine demethylase PHF8
42	SLC22A6	Q4U2R8	Solute carrier family 22 member 6
42	GPR18	Q14330	N-arachidonyl glycine receptor
42	TLR2	O60603	Toll-like receptor 2
42	FAAH	O00519	Fatty-acid amide hydrolase 1
42	SLC22A8	Q8TCC7	Solute carrier family 22 member 8
42	GABBR2	O75899	Gamma-aminobutyric acid type B receptor subunit 2
42	GABBR1	Q9UBS5	Gamma-aminobutyric acid type B receptor subunit 1
42	PTGIS	Q16647	Prostacyclin synthase
42	CNR1	P21554	Cannabinoid receptor 1
42	GABRR1	P24046	Gamma-aminobutyric acid receptor subunit rho-1
42	ABHD6	Q9BV23	Monoacylglycerol lipase ABHD6
42	ACE	P12821	Angiotensin-converting enzyme

42	ADAMTS5	Q9UNA0	ADAMTS5
42	AGTR1	P30556	Type-1 angiotensin II receptor (by homology)
42	AGTR2	P50052	Angiotensin II receptor
42	AKR1B1	P15121	Aldose reductase
42	ALOX5	P09917	Arachidonate 5-lipoxygenase
42	ALOX5AP	P20292	5-lipoxygenase activating protein
42	BMP1	P13497	Bone morphogenetic protein 1
42	CCKAR	P32238	Cholecystokinin A receptor
42	CCKBR	P32239	Cholecystokinin B receptor (by homology)
42	CDC25C	P30307	Dual specificity phosphatase Cdc25C
42	CMA1	P23946	Chymase
42	CPT1A	P50416	Carnitine O-palmitoyltransferase 1, liver isoform (by homology)
42	CPT1B	Q92523	Carnitine O-palmitoyltransferase 1, muscle isoform (by homology)
42	CTSS	P25774	Cathepsin S
42	CXCR2	P25025	Interleukin-8 receptor B
42	CYP26A1	O43174	Cytochrome P450 26A1
42	CYP26B1	Q9NR63	Cytochrome P450 26B1
42	DAGLA	Q9Y4D2	Sn1-specific diacylglycerol lipase alpha
42	EDNRA	P25101	Endothelin receptor ET-A
42	EDNRB	P24530	Endothelin receptor ET-B
42	ENPP2	Q13822	Autotaxin
42	EPHX1	P07099	Epoxide hydrolase 1
42	EPHX2	P34913	Epoxide hydratase
42	FABP2	P12104	Fatty acid binding protein intestinal
42	FABP3	P05413	Fatty acid binding protein muscle
42	FABP4	P15090	Fatty acid binding protein adipocyte
42	FDFT1	P37268	Squalene synthetase
42	FFAR1	O14842	Free fatty acid receptor 1
42	FFAR2	O15552	Free fatty acid receptor 2
42	FFAR4	Q5NUL3	G-protein coupled receptor 120
42	FNTA	P49354	Protein farnesyltransferase/geranylgeranyltransferase type-1 subunit alpha
42	FNTB	P49356	Protein farnesyltransferase subunit beta
42	GCG	P01275	Glucagon
42	GCGR	P47871	Glucagon receptor
42	GIPR	P48546	Gastric inhibitory polypeptide receptor
42	GLRA1	P23415	Glycine receptor subunit alpha-1
42	GRM2	Q14416	Metabotropic glutamate receptor 2 (by homology)
42	GYS1	P13807	Muscle glycogen synthase

42	HMGCR	P04035	HMG-CoA reductase
42	ITGAL	P20701	Leukocyte adhesion glycoprotein LFA-1 alpha
42	ITGAL	P20701	Integrin alpha-L
42	ICAM1	P05362	Intercellular adhesion molecule 1
42	ITGB2	P05107	Integrin beta-2
42	ITGAV	P06756	Integrin alpha-V
42	ITGB1	P05556	Integrin beta-1
42	ITGB3	P05106	Integrin beta-3
42	ITGB7	P26010	Integrin beta-7
42	KCNH2	Q12809	HERG
42	KDM2A	Q9Y2K7	Lysine-specific demethylase 2A
42	KEAP1	Q14145	Kelch-like ECH-associated protein 1
42	LDHA	P00338	L-lactate dehydrogenase A chain
42	LDHB	P07195	L-lactate dehydrogenase B chain
42	MCL1	Q07820	Induced myeloid leukemia cell differentiation protein Mcl-1
42	MDM2	Q00987	p53-binding protein Mdm-2
42	MME	P08473	Neprilysin
42	MMP1	P03956	Matrix metalloproteinase 1
42	MMP12	P39900	Matrix metalloproteinase 12
42	MMP13	P45452	Matrix metalloproteinase 13
42	MMP2	P08253	Matrix metalloproteinase 2
42	MMP3	P08254	Matrix metalloproteinase 3
42	MMP8	P22894	Matrix metalloproteinase 8
42	MMP9	P14780	Matrix metalloproteinase 9
42	NR1H2	P55055	LXR-beta
42	NR1H4	Q96RI1	Bile acid receptor FXR
42	NTSR1	P30989	Neurotensin receptor 1
42	OXER1	Q8TDS5	Oxoeicosanoid receptor 1
42	OXTR	P30559	Oxytocin receptor (by homology)
42	P2RY12	Q9H244	Purinergic receptor P2Y12
42	PDE4A	P27815	Phosphodiesterase 4A
42	PDE4B	Q07343	Phosphodiesterase 4B
42	PDE4D	Q08499	Phosphodiesterase 4D
42	PGR	P06401	Progesterone receptor
42	PLA2G10	O15496	Group X secretory phospholipase A2
42	PLA2G2A	P14555	Phospholipase A2 group IIA
42	POLB	P06746	DNA polymerase beta (by homology)
42	PPARA	Q07869	Peroxisome proliferator-activated receptor alpha
42	PPARD	Q03181	Peroxisome proliferator-activated receptor delta

42	PPARG	P37231	Peroxisome proliferator-activated receptor gamma
42	PRKAG1	P54619	5'-AMP-activated protein kinase subunit gamma-1
42	PRKAB1	Q9Y478	5'-AMP-activated protein kinase subunit beta-1
42	PRKAA2	P54646	5'-AMP-activated protein kinase catalytic subunit alpha-2
42	PSEN1	P49768	Presenilin 1
42	PSEN2	P49810	Presenilin-2
42	PSENEN	Q9NZ42	Gamma-secretase subunit PEN-2
42	NCSTN	Q92542	Nicastrin
42	APH1A	Q96BI3	Gamma-secretase subunit APH-1A
42	PSEN1	P49768	Presenilin-1
42	APH1B	Q8WW43	Gamma-secretase subunit APH-1B
42	PTGDR	Q13258	Prostanoid DP receptor
42	PTGDR2	Q9Y5Y4	G protein-coupled receptor 44
42	PTGER3	P43115	Prostanoid EP3 receptor
42	PTGES2	Q9H7Z7	Prostaglandin E synthase 2
42	RARA	P10276	Retinoic acid receptor alpha
42	RARB	P10826	Retinoic acid receptor beta
42	RARG	P13631	Retinoic acid receptor gamma
42	RBP4	P02753	Plasma retinol-binding protein
42	RXRA	P19793	Retinoid X receptor alpha
42	RXRB	P28702	Retinoid X receptor beta
42	RXRG	P48443	Retinoid X receptor gamma
42	S1PR1	P21453	Sphingosine 1-phosphate receptor Edg-1
42	SLC10A2	Q12908	Ileal bile acid transporter
42	SLC16A1	P53985	Monocarboxylate transporter 1 (by homology)
42	SRD5A1	P18405	Steroid 5-alpha-reductase 1
42	SRD5A2	P31213	Steroid 5-alpha-reductase 2
42	TBXA2R	P21731	Thromboxane A2 receptor
42	TBXAS1	P24557	Thromboxane-A synthase
42	TRPM8	Q7Z2W7	Transient receptor potential cation channel subfamily M member 8
42	TSPO	P30536	Translocator protein (by homology)
42	VCP	P55072	Transitional endoplasmic reticulum ATPase
43	CHRM2	P08172	Muscarinic acetylcholine receptor M2
43	CHRM3	P20309	Muscarinic acetylcholine receptor M3

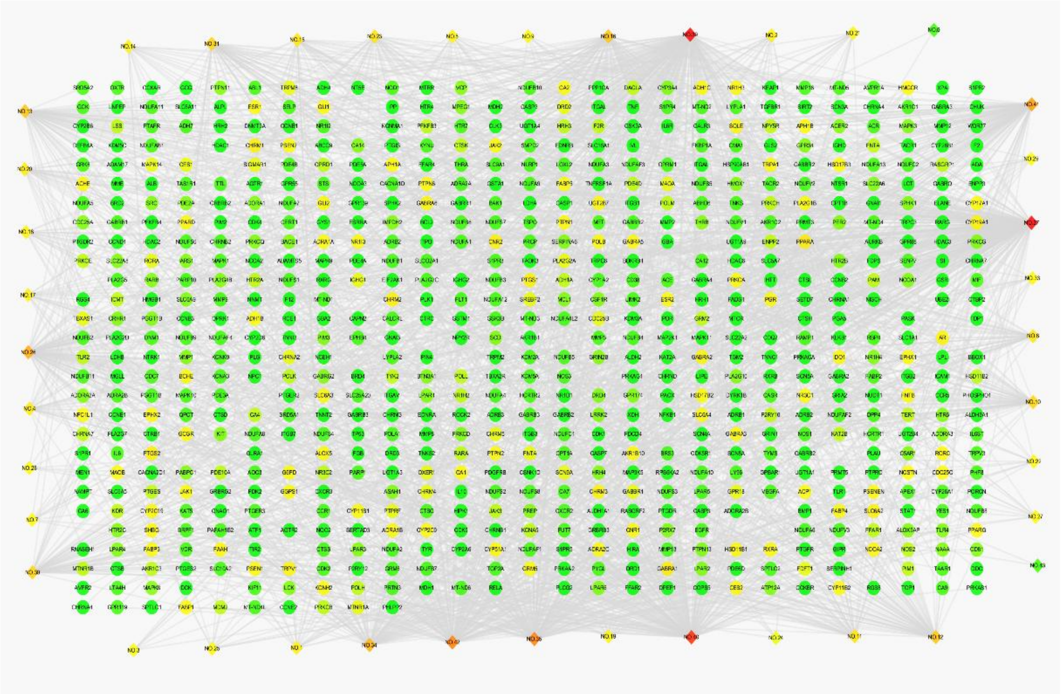

Figure S1 The component-target network of essential oil composition. The diamond nodes represent ingredients, and the circular nodes represent targets. The colors of the nodes are illustrated from red to yellow in descending order of degree values.
